# Long-term changes in adiposity markers during and after antidepressant therapy in a community cohort

**DOI:** 10.1038/s41398-024-03032-5

**Published:** 2024-08-13

**Authors:** Jessica Mwinyi, Marie-Pierre F. Strippoli, Sofia H. Kanders, Helgi B. Schiöth, Chin B. Eap, Aurélie M. Lasserre, Pedro Marques-Vidal, Caroline L. Vandeleur, Martin Preisig

**Affiliations:** 1 Department of Surgical Sciences, Uppsala University, Uppsala, Sweden; 2 Psychiatric Epidemiology and Psychopathology Research Center, Department of Psychiatry, Lausanne University Hospital and University of Lausanne, Prilly, Switzerland; 3 Centre for Clinical Research, Region Västmanland, Uppsala University, Uppsala, Sweden; 4 School of Pharmaceutical Sciences, University of Geneva, University of Lausanne, Geneva, Switzerland; 5 Center for Research and Innovation in Clinical Pharmaceutical Sciences, University of Lausanne, Lausanne, Switzerland; 6 Institute of Pharmaceutical Sciences of Western Switzerland, University of Geneva, University of Lausanne, Geneva, Switzerland; 7 Unit of Pharmacogenetics and Clinical Psychopharmacology, Centre for Psychiatric Neuroscience, Department of Psychiatry, Lausanne University Hospital and University of Lausanne, Prilly, Switzerland; 8 Addiction Medicine, Department of Psychiatry, Lausanne University Hospital and University of Lausanne, Prilly, Switzerland; 9 Internal Medicine, Department of Medicine, Lausanne University Hospital and University of Lausanne, Lausanne, Switzerland

**Keywords:** Depression, Physiology

## Abstract

Research on antidepressant-related weight changes over more than 12 months is scarce and adjustment for the effects of depressive episodes has rarely been applied. Accordingly, our aim was to assess the associations of the use of any antidepressants, subclasses of antidepressant and specific compounds prior to baseline and during a 5.5-year follow-up with changes in adiposity markers, and the effect of sex on these associations, with adjustment for multiple confounders including the effects of depressive episodes and their severity. Data stemmed from a prospective cohort study including 2479 randomly selected 35–66 year-old residents of an urban area (mean age 49.9 years, 53.3% women) who underwent physical and psychiatric evaluations at baseline and follow-up. Weight, height, waist circumference, and body fat were measured by trained nurses and information on diagnosis and antidepressant use prior to baseline and during follow-up was collected through standardized interviews. In the fully adjusted models, the number of antidepressants, mainly SSRIs and TCAs, used prior to baseline, was associated with a lower increase of body-mass index (BMI, β (95%CI) = −0.12 (−0.19, −0.05)) and waist circumference (β = −0.28 (−0.56, −0.01)), whereas participants treated with antidepressants during the follow-up had a steeper increase in BMI (β = 0.32 (0.13, 0.50)) and waist circumference (β = 1.23 (0.44, 2.01)). Within the class of SSRIs, the use of fluoxetine, sertraline or escitalopram during follow-up was associated with a steeper increase in adiposity markers. The associations of SSRIs with BMI and waist circumference were only observed when the SSRIs were used during the second period of the follow-up. Sex did not moderate these associations. Our findings suggest an increase of adiposity markers during sustained treatment with TCAs and SSRIs, which however return to normal levels after cessation of treatment. Hence, the benefit of long-term administration of these antidepressants should be carefully weighed against the potential risk of weight gain.

## Introduction

Major depressive disorder (MDD) is characterized by a complex co-morbidity profile including obesity [1–3]. Aside from poor health behavior, the effects of antidepressants (ADs) have been suggested to mediate the association between MDD and subsequent weight gain [4]. Considering the concomitant rise of prescriptions of ADs in the last decades [5–7], the potentially weight increasing effect of ADs has become a challenging public health issue.

Numerous randomized and open clinical trials as well as epidemiological studies have addressed this topic and suggest that the classical tricyclics (TCA) are associated with weight gain [8–11]. A meta-analysis of clinical trials, which could only identify 30 placebo-controlled randomized studies also providing information on weight gain during acute or maintenance treatment, has evidenced that among the assessed compounds only few were associated with weight changes [10]. The classical TCA amitriptyline, the noradrenergic and specific serotoninergic AD (NaSSA) mirtazapine and the selective serotonin reuptake inhibitor (SSRIs) paroxetine were associated with greater risk of weight gain, whereas the SSRI fluoxetine and the norepinephrine and dopamine reuptake inhibitor (NDRI) bupropion were associated with weight loss, although this effect of fluoxetine was limited to the 4–12 week’s acute treatment phase [10]. A more recent meta-analysis confirmed weight gain associated with the use of amitriptyline and mirtazapine and weight loss associated with the use of fluoxetine and bupropion [11]. However, although some of these studies did not only include acute but also maintenance treatment, they could not inform on associations between AD use of more than 12 months and weight changes. Indeed, the longest placebo-controlled clinical trial included in these metanalyses did not exceed a period of more than 12 months [11]. Accordingly, available information on weight changes associated with sustained AD use beyond this period relies entirely on prospective cohort studies. A systematic review of seven cohort studies involving follow-up periods between 2 and 18 years has documented at least 5% weight gain in individuals with AD use in most of the studies [12]. However, besides the risk of indication bias, which is inherent in all observational research, existing long-term studies are subject to several limitations including not systematically recording data on weight in primary care databases [13], using self-reported weight [14], and lack of assessment of depressive episodes or symptoms impeding distinction between the effects of ADs and those of depressive episodes [13]. Moreover, none of the previous observational studies could identify episodes with atypical characteristics, which have been shown to be most strongly associated with weight gain [15, 16]. Finally, most studies only assessed changes in weight or body mass index (BMI), although recent data have suggested that waist circumference is more strongly associated with the risk of cardiovascular disease than BMI [17] and that body fat percentage could be an independent risk factor for mortality [18].

Regarding potential differences in weight gain between men and women using AD, randomized clinical trials have hardly provided information to this question [19, 20], and also data from observational studies are scarce. In a naturalistic clinical follow-up study, women using ADs reported weight gain more frequently than men [21]. Similarly, a population-based study on older adults found measured weight gain in women who used SSRIs for longer than 90 days but not in men [22].

Taken together, the bulk of existing research suggests that TCAs are associated with weight gain [8–10], whereas the impact of SSRIs on weight changes is still controversial and may depend on the specific compound and the duration of treatment [10].

The aim of the present population-based study was to determine the prospective associations of the use of AD, subclasses of AD, and specific compounds prior to baseline and during a 5-year follow-up period on change of adiposity in terms of BMI, waist circumference and fat mass during this follow-up as well as the effect of sex on these associations, adjusting for multiple potential confounders including socio-demographic and behavioral factors, adiposity markers at baseline, early trauma, the use of other potential weight-inducing drugs, anxiety and substance use disorders, as well as subtypes of major depressive episodes (MDE: atypical, melancholic, unspecified) that occurred prior to baseline and those occurring during the follow-up. In order to mitigate the risk of indication bias inherent in naturalistic research, (e.g., ADs are likely to be prescribed in more severe forms of MDD, which may be associated with elevated weight gain), analyses were also adjusted for multiple depression severity markers. Given that the observation period included an intermediate assessment of drug prescriptions, we could separately assess the associations of (1) AD prescription over the whole follow-up period, (2) prescription that occurred exclusively prior to the intermediate assessment, and (3) prescription that occurred exclusively after the intermediate assessment with change of adiposity markers during this follow-up.

## Material and methods

### Cohort and participants

Analyses were performed on data from CoLaus|PsyCoLaus [23, 24], a prospective follow-up study designed to assess the associations between mental disorders and cardiovascular risk factors in the community. A total of 6734 individuals aged 35–75 years were randomly selected from the residents of the city of Lausanne, Switzerland, between 2003 and 2006 according to the civil register. Sixty-seven percent of the 35–66-year-old participants of the physical baseline exam (*n* = 5535) also accepted the psychiatric evaluation, leading to a sample size of 3719 participants with both somatic and psychiatric baseline assessments. Participants with a diagnosis of bipolar or schizoaffective disorder, schizophrenia or eating disorder were excluded from the present analyses given that these disorders are likely to be associated with metabolic changes. Moreover, we could not include the 7.8% non-White participants because medication data were not collected for them at baseline. Among the remaining 3270 participants, 43 died during the follow-up (mean (SD) duration 5.5 (0.4) years) and 2479 accepted both the physical and psychiatric follow-up evaluations (76.8% participation among survivors, flow diagram in Fig. S1*in Supplement*). Non-participants at follow-up were more likely than participants to have lower socioeconomic status, to live alone, to be less physically active, and to be current smokers.

### Ethics approval and consent to participate

The institutional Ethics Committee for Clinical Research of the Medical and Biological Faculty of the University of Lausanne, which afterward became the Ethics Committee of the Canton of Vaud for human research (www.cer-vd.ch), approved the CoLaus|PsyCoLaus project (reference 239/09). The study was performed in agreement with the Helsinki Declaration and its former amendments and in accordance with the applicable Swiss legislation. All participants signed a written informed consent before the evaluations.

### Assessments

Physical measures were taken in identical ways at the baseline and the follow-up visits. Body weight and height were measured with participants standing without shoes in light indoor clothes. Weight was measured after fasting for 8 h. Waist circumference was determined using a non-stretchable tape over the unclothed abdomen at the mid-point between the lowest rib and the iliac crest, and the average of two measurements was used for analyses. Fat mass was assessed by bio impedance [25] using the Bodystat 1500 analyzer.

Information on medication, sociodemographic characteristics and health-related behaviors including smoking, alcohol consumption (number of standard drinks per week) and physical activity, was collected through standardized interviews. At the physical evaluations, regular drug treatment was assessed for the preceding 6 months. Participants were requested to present any drug prescriptions for this period. Additional information on psychotropic treatment was elicited at the psychiatric baseline evaluation for the most severe and the most recent MDE and at the psychiatric follow-up evaluation for the most severe MDE since the previous baseline evaluation (Fig. S2*in Supplement*). Accordingly, information on regular AD use was collected four times (at the physical and the psychiatric baseline evaluations as well as at the physical and psychiatric follow-up evaluations). This allowed us to distinguish between three different periods of AD use (a) prior to the physical baseline; (b) physical baseline to psychiatric baseline (“first follow-up period”; mean (SD) duration = 1.23 (0.38) years); (c) psychiatric baseline to physical follow-up (“second follow-up period”; mean (SD) duration = 4.28 (0.56) years). Hence, with respect to AD use within the follow-up period, we could subdivide the users into those who only used ADs during the first period of the follow-up vs. those who used ADs during the second period of the follow-up vs. those who used ADs during the two periods of the follow-up. ADs were coded using the ATC system and categorized into four groups: (1) TCAs, (2) SSRIs, (3) mirtazapine or trazodone, and (4) others including serotonin-noradrenaline reuptake inhibitors (SNRIs), NDRI, and Monoamine oxidase inhibitors (MAOI) (definitions and distributions in Table S1*in Supplement*).

The level of socioeconomic status (SES) was determined according to the Hollingshead scale [26]. Participants were considered to be physically inactive in the case of physical activity reported for less than 20 min twice a week.

Diagnostic information on mental disorders at baseline and follow-up was collected using the French version [27] of the semi-structured Diagnostic Interview for Genetic Studies (DIGS) [28], which revealed adequate inter-rater and test-retest reliability for psychotic, mood and substance use disorders [29, 30]. The DIGS was completed with the PTSD and anxiety disorder sections of the Schedule for Affective Disorders and Schizophrenia - Lifetime Version (SADS-L) [31, 32]. Diagnoses of psychiatric lifetime disorders and depressive episodes during follow-up were assigned according to the DSM-IV [33]. MDD was subdivided according to the lifetime history of episodes with atypical or melancholic features according to the DSM-IV specifiers into three subtypes: (1) MDD with atypical features only, (2) MDD with melancholic features only, (3) unspecified MDD with neither atypical nor melancholic features, or with both atypical and melancholic features. The DIGS also assesses the timing and duration of depressive episodes, the number of symptoms during episodes, and the impact on psychosocial functioning (global assessment of functioning (GAF) scores). The information collected on episodes also included the type of treatment (inpatient vs. outpatient), the occurrence of psychotic features, suicidal ideas or behavior, and the duration of episodes allowing us to compute the participant’s time spent in episodes during the follow-up. The lifetime history of MDD in first-degree family members was systematically assessed using the Family History-Research Diagnostic Criteria (FH-RDC) [34]. The PTSD section of the interview also assesses early physical or sexual abuse. Interviewers were psychologists, who were trained over at least a 1-month period. Each interview was reviewed by an experienced senior psychologist.

### Statistical analysis

Analyses were performed using the Statistical Analysis System (SAS, Cary, NC, USA), version 9.4, for Windows. In order to assess the effect of sex on the associations of any AD use, subclasses of AD, and specific compounds prior to baseline and during follow-up with changes in adiposity markers (BMI, waist circumference, fat mass) between baseline and follow-up, we first tested interactions between sex and subtypes of AD use prior to baseline and during the follow-up. Associations of AD use with changes in adiposity markers between baseline and follow-up were established using serially adjusted robust multiple regression models as residuals did not reveal a normal distribution. The first model (Model 1) was adjusted for socio-demographic characteristics (sex, age, SES, living alone during follow-up), early physical or sexual abuse (prior to the age of 18 years), baseline levels of the corresponding adiposity marker, health-related behavioral characteristics (physical inactivity, alcohol drinks per week, smoking status) during follow-up, the occurrence of anxiety disorders or illicit drug dependence during follow-up, the use of possibly weight gain inducing drugs (other than ADs) during follow-up (list and details on the list extraction procedure in Table S2*in Supplement*), and the length of follow-up. Including the same adjustments as for the previous model, Model 2 was further adjusted for the lifetime history of MDD subtypes at baseline, the occurrence of subtypes of MDE during the follow-up, and current vs. remitted MDD status at the physical follow-up. Given that the likelihood of AD treatment may be related to the severity of MDE, Model 3 was further adjusted for MDD severity markers in terms of number of symptoms during the most severe episode, time spent in MDE and the GAF score during follow-up, hospitalization, psychotic features, and suicidality during follow-up, and a positive family history for MDD. Complementary analyses were performed to assess associations between the timing of AD use during the follow-up (first period only vs. second period only vs. both first and second period of follow-up). Alternative analyses were conducted using a propensity score for adjustment for potential confounders. The propensity score was estimated using a logistic regression model in which lifetime treatment status (treatment vs. not) was regressed on the same covariate adjustment that was made to Model 3. Once the propensity score has been estimated, we used it as a covariate adjustment. Finally, in order to establish whether AD use was associated with a clinically significant increase of adiposity markers, analyses were conducted using the proportion of participants with a 5% increase of adiposity markers during the follow-up as the outcome variable.

## Results

Table 1 provides the description of the whole sample and those of the subsamples according to AD medication during follow-up. Given that a participant could have used more than one AD during follow-up the groupings are non-exclusive. Indeed, among the 406 participants (16.4% of the sample) who were treated with ADs during the follow-up, 321 used one compound, 61 two compounds, and 24 three compounds or more, with 62 participants using compounds of at least two different classes of ADs. Among those who used ADs, 13% used TCAs, 69% SSRIs, 12% mirtazapine or trazodone and 20% others. Approximately 3% of the whole sample used ADs either exclusively during the shorter first period or during the entire follow-up, whereas more than 10% used ADs exclusively during the longer second period of the follow-up. Nearly a sixth of the total sample was already treated with at least one AD prior to baseline. Among the 40% of the participants with a lifetime MDD at baseline, the majority met criteria for unspecified MDD, followed by MDD with melancholic features and MDD with atypical features. One out of five participants developed a MDE during the follow-up period.

**Table 1:** Characteristics of participants.

	Antidepressants during follow-up						
	Whole sample	Any	TCA	SSRI	Mirtazapine/Trazodone	Other^a^	None
	(*n* = 2479)	(*n* = 406)	(*n* = 53)	(*n* = 279)	(*n* = 47)	(*n* = 82)	(*n* = 2073)
	No. (%)/mean (SD)	No. (%)/mean (SD)	No. (%)/mean (SD)	No. (%)/mean (SD)	No. (%)/mean (SD)	No. (%)/mean (SD)	No. (%)/mean (SD)
Socio-demographic characteristics							
Age at baseline [years], mean (SD)	49.9 (8.8)	49.6 (8.6)	51.6 (8.7)	49.2 (8.4)	49.4 (8.8)	48.8 (8.3)	49.9 (8.9)
Men, No. (%)	1158 (46.7)	132 (32.5)	19 (35.8)	85 (30.5)	19 (40.4)	30 (36.6)	1026 (49.5)
Socio-economic status at baseline^b^, mean (SD)	3.4 (1.2)	3.3 (1.2)	3.2 (1.1)	3.3 (1.2)	3.4 (1.2)	3.4 (1.2)	3.4 (1.2)
Living alone during follow-up, No. (%)	486 (19.7)	96 (23.7)	15 (28.3)	70 (25.2)	13 (27.7)	10 (12.2)	390 (18.9)
Length of follow-up [years], mean (SD)	5.5 (0.4)	5.5 (0.4)	5.5 (0.4)	5.5 (0.4)	5.5 (0.4)	5.5 (0.2)	5.5 (0.4)
Early trauma							
Early physical or sexual abuse, No. (%)	39 (1.6)	8 (2.0)	2 (3.8)	6 (2.2)	0 (0.0)	3 (3.7)	31 (1.5)
Antidepressants prior to baseline							
Number of antidepressant compounds, mean (SD)	0.2 (0.5)	0.6 (0.7)	0.9 (1.0)	0.7 (0.7)	0.7 (1.0)	0.8 (0.9)	0.1 (0.3)
Antidepressants, No. (%)							
Any	377 (15.2)	217 (53.4)	33 (62.3)	162 (58.1)	24 (51.1)	48 (58.5)	160 (7.7)
TCA	76 (3.1)	40 (9.9)	24 (45.3)	17 (6.1)	5 (10.6)	11 (13.4)	36 (1.7)
SSRI	280 (11.3)	164 (40.4)	14 (26.4)	147 (52.7)	14 (29.8)	25 (30.5)	116 (5.6)
Mirtazapine/Trazodone	24 (1.0)	14 (3.4)	3 (5.7)	8 (2.9)	7 (14.9)	3 (3.7)	10 (0.5)
Other^a^	41 (1.7)	27 (6.7)	3 (5.7)	9 (3.2)	5 (10.6)	23 (28.0)	14 (0.7)
Medication during follow-up							
Antidepressants, No. (%)							
At any time during the follow-up	406 (16.4)	406 (100.0)	53 (100.0)	279 (100.0)	47 (100.0)	82 (100.0)	-
During first period of follow-up only	69 (2.8)	69 (17.0)	14 (26.4)	46 (16.5)	6 (12.8)	9 (11.0)	-
During second period of follow-up only	250 (10.1)	250 (61.6)	34 (64.2)	194 (69.5)	35 (74.5)	59 (72.0)	-
During first and second periods of follow-up	87 (3.5)	87 (21.4)	5 (9.4)	39 (14.0)	6 (12.8)	14 (17.1)	-
Weight-gain-inducing drug, No. (%)	196 (7.9)	52 (12.8)	6 (11.3)	37 (13.3)	3 (6.4)	11 (13.4)	144 (6.9)
Behavioral factors during follow-up							
Physically inactive^c^, No. (%)	811 (32.7)	156 (38.4)	21 (39.6)	107 (38.4)	16 (34.0)	35 (42.7)	655 (31.6)
Smoking status, No. (%)							
Current	745 (30.1)	155 (38.2)	18 (34.0)	108 (38.7)	21 (44.7)	35 (42.7)	590 (28.5)
Former	798 (32.2)	118 (29.1)	17 (32.1)	79 (28.3)	10 (21.3)	22 (26.8)	680 (32.8)
Never	936 (37.8)	133 (32.8)	18 (34.0)	92 (33.0)	16 (34.0)	25 (30.5)	803 (38.7)
Number of alcoholic drinks per week, mean (SD)	6.8 (7.9)	6.2 (8.1)	4.5 (5.6)	6.1 (8.2)	6.3 (6.6)	7.4 (10.0)	6.9 (7.8)
Family history of mood disorders							
Major depressive disorder, No. (%)	1124 (48.2)	217 (56.5)	32 (61.5)	149 (27.3)	26 (57.8)	54 (67.5)	907 (46.5)
History of psychiatric disorders at baseline							
Major depressive disorder, No. (%)							
Any	1090 (44.0)	328 (80.8)	40 (75.5)	230 (82.4)	37 (78.7)	72 (87.8)	762 (36.8)
Atypical	165 (6.7)	59 (14.5)	4 (7.5)	48 (17.2)	4 (8.5)	10 (12.2)	106 (5.1)
Melancholic	320 (12.9)	111 (27.3)	19 (35.8)	71 (25.4)	13 (27.7)	28 (34.1)	209 (10.1)
Unspecified	605 (24.4)	158 (38.9)	17 (32.1)	111 (39.8)	20 (42.6)	34 (41.5)	447 (21.6)
None	1389 (56.0)	78 (29.2)	13 (24.5)	49 (17.6)	10 (21.3)	10 (12.2)	1311 (63.2)
Psychiatric disorders during follow-up							
Major depressive episode, No. (%)							
Any	493 (19.9)	196 (48.3)	25 (47.2)	139 (49.8)	28 (59.6)	54 (65.9)	297 (14.3)
Atypical	96 (3.9)	38 (9.4)	7 (13.2)	25 (9.0)	3 (6.4)	14 (17.1)	58 (2.8)
Melancholic	119 (4.8)	53 (13.1)	4 (7.5)	39 (14.0)	9 (19.1)	15 (18.3)	66 (3.2)
Unspecified	278 (11.2)	105 (25.9)	14 (26.4)	75 (26.9)	16 (34.0)	25 (30.5)	173 (8.3)
None	1986 (80.1)	210 (51.7)	28 (52.8)	140 (50.1)	19 (40.4)	28 (34.1)	1776 (85.7)
Current MDE at follow-up, No. (%)	307 (12.4)	136 (33.5)	16 (30.2)	104 (37.3)	19 (40.4)	35 (42.7)	171 (8.2)
Number of symptoms of most severe MDE, mean (SD)	1.3 (2.7)	3.4 (3.6)	3.3 (3.6)	3.6 (3.7)	4.7 (3.6)	4.3 (3.7)	0.9 (2.3)
Time spent in MDE (weeks), mean (SD)	18.6 (82.7)	59.0 (140.7)	66.8 (155.6)	58.5 (128.4)	99.8 (182.5)	59.5 (101.4)	10.7 (62.6)
Suicidality, No. (%)	209 (8.4)	106 (26.1)	13 (24.5)	78 (28.0)	15 (31.9)	28 (34.2)	103 (5.0)
Hospitalization, No. (%)	18 (0.7)	17 (4.2)	1 (1.9)	14 (5.0)	4 (8.5)	7 (8.5)	1 (0.1)
Psychotic features, No. (%)	14 (0.6)	11 (2.7)	0 (0.0)	11 (3.9)	2 (4.3)	2 (2.4)	3 (0.1)
GAF score, mean (SD)	82.1 (16.4)	69.2 (22.4)	68.7 (23.2)	68.4 (22.7)	60.1 (22.9)	63.9 (23.0)	84.6 (13.6)
Illicit drug dependence^d^, No. (%)	7 (0.3)	4 (1.0)	0 (0.0)	3 (1.1)	1 (2.1)	1 (1.2)	3 (0.1)
Anxiety disorders^e^, No. (%)	163 (6.6)	61 (15.0)	6 (11.3)	45 (16.1)	11 (23.4)	13 (15.9)	102 (4.9)
Adiposity markers at baseline							
Body Mass Index [kg/m^2^], mean (SD)	25.3 (4.4)	25.4 (4.8)	24.1 (3.7)	25.6 (5.0)	25.6 (4.8)	25.1 (4.0)	25.3 (4.3)
Waist [cm], mean (SD)	87.7 (13.0)	87.0 (13.2)	83.6 (10.9)	87.1 (13.6)	89.2 (13.6)	86.1 (11.6)	87.8 (13.0)
Fat mass [%], mean (SD)	28.1 (8.7)	30.1 (8.7)	28.7 (7.4)	30.5 (9.0)	29.0 (7.6)	29.4 (7.3)	27.6 (8.7)
Adiposity markers at follow-up							
Body Mass Index [kg/m^2^], mean (SD)	25.9 (4.6)	26.3 (5.3)	25.2 (4.7)	26.6 (5.5)	26.0 (4.8)	25.9 (4.5)	25.9 (4.4)
Waist [cm], mean (SD)	91.1 (13.0)	91.6 (14.0)	89.8 (12.2)	91.8 (14.3)	92.0 (12.1)	91.4 (13.4)	91.0 (12.8)
Fat mass [%], mean (SD)	30.0 (8.7)	32.6 (8.9)	31.1 (8.4)	33.2 (9.3)	31.1 (7.3)	31.5 (7.6)	29.5 (8.6)

*TCA* Tricyclic antidepressants, *SSRI* Selective Serotonin Reuptake Inhibitors, *SD* standard deviation, *MDE* major depressive episode, *GAF* global assessment of functioning.

^a^SNRI (Serotonin-Noradrenaline Reuptake Inhibitors)/NDRI (Noradrenaline Reuptake Inhibitors)/MAOI(Monoamine Oxidase Inhibitors).

^b^According to the Hollingshead Scale (a value of 3 represents a level III (middle class)).

^c^Physically active less than 20 min twice a week.

^d^Dependence on cannabis, cocaine, stimulant, sedative, or hallucinogen.

^e^Generalized anxiety disorder, social phobia, panic disorder, or agoraphobia.

Using robust multiple regression models, we first tested for interactions between sex and AD use prior to baseline or during the follow-up regarding changes of adiposity markers between men and women. However, none of the tested interactions terms reached the level of statistical significance. Hence, we only provide the results for the whole cohort.

Table 2 presents the change in adiposity markers by use of any AD during follow-up and the results of the serially adjusted models. According to the fully adjusted Model 3, which also accounted for the occurrence and severity of MDE, the number of AD used prior to baseline was associated with a lower increase in BMI and waist circumference, whereas treatment with AD during follow-up was associated with a steeper increase in these adiposity markers. There was no association with fat mass.

**Table 2:** Change of adiposity markers during follow-up by any antidepressant use prior to baseline and during follow-up.

	Change in adiposity markers during follow-up								
	Crude change			Model 1		Model 2		Model 3	
	Mean (SD)	β^a^	(95%CI)	β	(95%CI)	β	(95%CI)	β	(95%CI)
Body Mass Index [kg/m^2^] (*n* = 2462)									
Antidepressants prior to baseline									
Number of compounds	—	−0.01	(−0.07, 0.04)	−0.08*	**(−0.14, −0.02)**	−0.12***	**(−0.18, −0.06)**	−0.12***	**(−0.19, −0.05)**
Antidepressants during follow-up									
Any antidepressants	0.9 (2.2)	0.35***	**(0.19, 0.50)**	0.42***	**(0.25, 0.59)**	0.34***	**(0.16, 0.52)**	0.32***	**(0.13, 0.50)**
No antidepressants (ref.)	0.6 (1.7)	0 (ref.)	—	0 (ref.)	—	0 (ref.)	—	0 (ref.)	—
Waist circumference [cm] (*n* = 2475)									
Antidepressants prior to baseline									
Number of compounds	—	0.06	(−0.19, 0.30)	−0.19	(−0.46, 0.08)	−0.28	(−0.56, 0.00)	−0.28*	**(−0.56, −0.01)**
Antidepressants during follow-up									
Any antidepressants	4.6 (7.3)	1.39***	**(0.73, 2.05)**	1.52***	**(0.79, 2.25)**	1.29**	**(0.52, 2.06)**	1.23**	**(0.44, 2.01)**
No antidepressants (ref.)	3.2 (6.6)	0 (ref.)	—	0 (ref.)	—	0 (ref.)	—	0 (ref.)	—
Fat mass [%] (*n* = 2079)									
Antidepressants prior to baseline									
Number of compounds	—	−0.01	(−0.18, 0.17)	−0.04	(−0.23, 0.15)	−0.11	(−0.30, 0.09)	−0.09	(−0.29, 0.11)
Antidepressants during follow-up									
Any antidepressants	2.5 (5.8)	0.37	(−0.10, 0.84)	0.39	(−0.13, 0.91)	0.30	(−0.25, 0.84)	0.18	(−0.38, 0.75)
No antidepressants (ref.)	2.1 (5.7)	0 (ref.)	—	0 (ref.)	—	0 (ref.)	—	0 (ref.)	—

Model 1 adjusted for socio-demographic characteristics (sex, age, socio-economic status, living alone during follow-up), early physical and sexual abuse, adiposity marker levels at baseline, behavioral factors (physical inactivity, smoking status, number of alcohol drinks per week) during follow-up, anxiety disorders and illicit drug dependence during follow-up, possibly weight gain inducing medication (other than antidepressants) during follow-up, and length of follow-up.

Model 2 = model 1 additionally adjusted for major depressive disorder (MDD) subtypes at baseline and during follow-up and current vs. remitted MDD status at follow-up.

Model 3 = model 2 additionally adjusted for severity during follow-up (number of symptoms of most severe major depressive episode (MDE), time spent in MDE, global assessment functioning (GAF) score, suicidality, hospitalization, psychotic features), and relatives with MDD.

*SD* standard deviation, *95%CI* 95% confidence interval. *ref*. reference group.

^a^Each model adjusted for age, sex, and adiposity marker at baseline.

* *p* < 0.05, ** *p* < 0.01, *** *p* < 0.001.

Significant results are indicated in bold.

Table 3 depicts the change in adiposity markers by AD classes during follow-up. The fully adjusted Model 3 revealed that participants treated with TCAs or SSRIs prior to baseline had a lower increase in BMI, whereas those treated with SSRIs had a steeper increase in BMI during follow-up than the other participants. Similarly, treatment with SSRIs prior to baseline was associated with a lower increase in waist circumference, whereas treatment with TCA or SSRIs during follow-up was associated with a steeper increase in waist circumference. Finally, the association between SSRI treatment during follow-up and a steeper increase in fat mass no longer reached the level of statistical significance after adjustment for the severity of MDE.

**Table 3:** Change of adiposity markers during follow-up by use of antidepressant class prior to baseline and during follow-up.

	Change in adiposity markers during follow-up								
	Crude change			Model 1		Model 2		Model 3	
	Mean (SD)	β^a^	(95%CI)	β	(95%CI)	β	(95%CI)	β	(95%CI)
Body Mass Index [kg/m^2^] (*n* = 2462)									
Antidepressants prior to baseline									
TCA	0.4 (1.9)	−0.25	(−0.58, 0.09)	−0.44*	**(−0.80, −0.07)**	−0.49**	**(−0.85, −0.13)**	−0.49**	**(−0.85, −0.13)**
SSRI	0.6 (2.3)	0.06	(−0.13, 0.24)	−0.15	(−0.35, 0.06)	−0.27*	**(−0.48, −0.06)**	−0.25*	**(−0.46, −0.04)**
Mirtazapine/Trazodone	1.2 (2.5)	−0.06	(−0.65, 0.52)	−0.06	(−0.65, 0.54)	−0.12	(−0.72, 0.47)	−0.13	(−0.72, 0.47)
Other^b^	1.0 (2.2)	−0.01	(−0.46, 0.44)	−0.22	(−0.70, 0.26)	−0.30	(−0.78, 0.18)	−0.27	(−0.76, 0.21)
No antidepressants (ref.)	0.6 (1.7)	0 (ref.)	—	0 (ref.)	—	0 (ref.)	—	0 (ref.)	—
Antidepressants during follow-up									
TCA	1.0 (1.9)	0.32	(−0.08, 0.72)	0.44*	**(0.01, 0.87)**	0.36	(−0.07, 0.79)	0.35	(−0.08, 0.77)
SSRI	1.0 (2.1)	0.36***	**(0.18, 0.55)**	0.42***	**(0.21, 0.62)**	0.36***	**(0.15, 0.57)**	0.34**	**(0.13, 0.56)**
Mirtazapine/Trazodone	0.4 (3.1)	−0.14	(−0.56, 0.28)	−0.08	(−0.51, 0.35)	−0.07	(−0.50, 0.36)	−0.10	(−0.53, 0.33)
Other^b^	0.9 (1.9)	0.16	(−0.16, 0.48)	0.31	(−0.05, 0.66)	0.26	(−0.09, 0.62)	0.22	(−0.15, 0.58)
No antidepressants (ref.)	0.6 (1.7)	0 (ref.)	—	0 (ref.)	—	0 (ref.)	—	0 (ref.)	—
Waist circumference [cm] (*n* = 2475)									
Antidepressants prior to baseline									
TCA	4.1 (8.0)	0.46	(−0.97, 1.89)	−0.35	(−1.86, 1.17)	−0.25	(−1.77, 1.27)	−0.26	(−1.79, 1.26)
SSRI	3.4 (7.4)	0.04	(−0.74, 0.83)	−0.71	(−1.59, 0.16)	−1.02*	**(**−**1.91**, −**0.12)**	−0.97*	**(**−**1.87**, −**0.08)**
Mirtazapine/Trazodone	2.9 (8.0)	−0.96	(−3.47, 1.55)	−1.14	(−3.68, 1.40)	−1.56	(−4.10, 0.98)	−1.84	(−4.40, 0.72)
Other^b^	4.2 (6.5)	0.72	(−1.20, 2.64)	−0.23	(−2.29, 1.83)	−0.35	(−2.41, 1.72)	−0.16	(−2.23, 1.91)
No antidepressants (ref.)	3.4 (6.6)	0 (ref.)	—	0 (ref.)	—	0 (ref.)	—	0 (ref.)	—
Antidepressants during follow-up									
TCA	6.2 (6.5)	2.42**	**(0.73, 4.12)**	2.52**	**(0.72, 4.32)**	2.28*	**(0.48, 4.09)**	2.16*	**(0.36, 3.97)**
SSRI	4.7 (7.3)	1.13**	**(0.34, 1.91)**	1.36**	**(0.48, 2.24)**	1.20**	**(0.29, 2.10)**	1.12*	**(0.19, 2.04)**
Mirtazapine/Trazodone	2.7 (8.4)	−0.28	(−2.07, 1.52)	−0.05	(−1.87, 1.78)	−0.05	(−1.89, 1.79)	−0.12	(−1.97, 1.73)
Other^b^	5.3 (6.6)	0.95	(−0.43, 2.34)	1.31	(−0.20, 2.81)	1.16	(−0.36, 2.68)	1.00	(−0.54, 2.55)
No antidepressants (ref.)	3.2 (6.6)	0 (ref.)	—	0 (ref.)	—	0 (ref.)	–	0 (ref.)	—
Fat mass [%] (*n* = 2079)									
Antidepressants prior to baseline									
TCA	1.4 (5.7)	−0.12	(−1.12, 0.88)	0.13	(−0.94, 1.20)	0.03	(−1.05, 1.10)	0.08	(−1.00, 1.17)
SSRI	2.2 (6.3)	0.43	(−0.12, 0.98)	0.23	(−0.38, 0.84)	0.06	(−0.56, 0.69)	0.12	(−0.51, 0.75)
Mirtazapine/Trazodone	0.8 (6.3)	−0.43	(−2.29, 1.43)	−0.28	(−2.15, 1.60)	−0.35	(−2.23, 1.53)	−0.37	(−2.26, 1.52)
Other^b^	2.2 (4.9)	−0.86	(−2.15, 0.44)	−0.86	(−2.26, 0.54)	−1.01	(−2.41, 0.40)	−0.95	(−2.37, 0.47)
No antidepressants (ref.)	2.2 (5.7)	0 (ref.)	—	0 (ref.)	—	0 (ref.)	—	0 (ref.)	—
Antidepressants during follow-up									
TCA	2.4 (6.1)	−0.27	(−1.42, 0.89)	−0.52	(−1.77, 0.72)	−0.65	(−1.89, 0.60)	−0.69	(−1.94, 0.57)
SSRI	2.7 (6.0)	0.89**	**(0.32, 1.46)**	0.73*	**(0.10, 1.36)**	0.70*	**(0.05, 1.35)**	0.61	(−0.05, 1.28)
Mirtazapine/Trazodone	2.5 (5.4)	−0.15	(−1.47, 1.17)	0.04	(−1.29, 1.37)	0.21	(−1.13, 1.55)	0.08	(−1.27, 1.43)
Other^b^	2.0 (4.0)	−0.52	(−1.45, 0.40)	−0.21	(−1.22, 0.80)	−0.35	(−1.38, 0.67)	−0.47	(−1.52, 0.58)
No antidepressants (ref.)	2.1 (5.7)	0 (ref.)	—	0 (ref.)	—	0 (ref.)	—	0 (ref.)	—

Model 1 adjusted for socio-demographic characteristics (sex, age, socio-economic status, living alone during follow-up), early physical and sexual abuse, adiposity marker levels at baseline, behavioral factors (physical inactivity, smoking status, number of drinks per week) during follow-up, anxiety disorders and illicit drug dependence during follow-up, possibly weight gain inducing medication (other than antidepressants) during follow-up, and length of follow-up.

Model 2 = model 1 additionally adjusted for major depressive disorder (MDD) subtypes at baseline and during follow-up and current vs. remitted MDD status at follow-up.

Model 3 = model 2 additionally adjusted for severity during follow-up (number of symptoms of most severe major depressive episode (MDE), time spent in MDE, global assessment functioning (GAF) score, suicidality, hospitalization, psychotic features), and relatives with MDD.

*TCA* Tricyclic antidepressants, *SSRI* Selective Serotonin Reuptake Inhibitors, *SD* standard deviation, *95%CI* 95% confidence interval. *ref*. reference group.

^a^Each model adjusted for age, sex and adiposity marker at baseline.

^b^SNRI (Serotonin-Noradrenaline Reuptake Inhibitors)/NDRI (Noradrenaline Reuptake Inhibitors)/MAOI(Monoamine Oxidase Inhibitors).

* *p* < 0.05, ** *p* < 0.01, *** *p* < 0.001.

Significant results are indicated in bold.

Among the specific compounds (Table 4), after multiple adjustments including use of AD prior to baseline, occurrence of MDE prior to baseline and during follow-up as well as depression severity, use of fluoxetine during follow-up was associated with a steeper increase in all three adiposity markers, use of sertraline with a steeper increase in BMI only, and use of escitalopram with a steeper increase in BMI and waist circumference.

**Table 4:** Change of adiposity markers during follow-up by use of specific antidepressants during follow-up.

	Change in adiposity markers during follow-up					
	Body Mass Index [kg/m^2^] (*n* = 2462)		Waist circumference [cm] (*n* = 2475)		Fat mass [%] (*n* = 2079)	
	β^a^	(95%CI)	β^a^	(95%CI)	β^a^	(95%CI)
TCA						
Clomipramine	−0.38	(−1.30, 0.53)	0.44	(−3.29, 4.17)	−0.94	(−3.49, 1.61)
Amitriptyline	0.10	(−0.54, 0.74)	1.63	(−1.10, 4.37)	−0.37	(−2.19, 1.45)
Melitracen and psycholeptics	0.41	(−0.33, 1.16)	1.70	(−1.48, 4.87)	0.33	(−1.98, 2.64)
SSRI						
Fluoxetine	0.37*	**(0.02, 0.72)**	1.88*	**(0.40, 3.36)**	1.54**	**(0.49, 2.58)**
Citalopram	−0.16	(−0.50, 0.19)	−0.33	(−1.79, 1.13)	−0.35	(−1.43, 0.73)
Paroxetine	−0.02	(−0.49, 0.44)	−0.69	(−2.67, 1.29)	0.81	(−0.63, 2.24)
Sertraline	0.65*	**(0.13, 1.16)**	1.23	(−0.96, 3.41)	1.36	(−0.18, 2.90)
Escitalopram	0.55***	**(0.23, 0.87)**	1.95**	**(0.57, 3.33)**	0.64	(−0.38, 1.65)
Mirtazapine/Trazodone						
Mirtazapine	−0.26	(−0.79, 0.27)	−1.18	(−3.44, 1.09)	0.34	(−1.39, 2.07)
Trazodone	0.36	(−0.36, 1.07)	1.53	(−1.54, 4.60)	−0.63	(−2.76, 1.50)
SNRI						
Venlafaxine	0.24	(−0.18, 0.67)	0.83	(−0.99, 2.65)	−0.50	(−1.73, 0.73)
Duloxetine	0.24	(−0.28, 0.75)	0.90	(−1.32, 3.12)	−0.57	(−2.06, 0.92)
No antidepressants (ref.)	0 (ref.)	—	0 (ref.)	—	0 (ref.)	—

*TCA* tricyclic antidepressants, *SSRI* selective serotonin reuptake inhibitors, *SNRI* serotonin-noradrenaline reuptake inhibitors, *95%CI* 95% confidence interval. *ref*. reference group.

^a^Adjusted for socio-demographic characteristics (sex, age, socio-economic status, living alone during follow-up), early physical and sexual abuse, adiposity marker levels at baseline, behavioral factors (physical inactivity, smoking status, number of drinks per week) during follow-up, anxiety disorders and illicit drug dependence during follow-up, possibly weight gain inducing medication (other than antidepressants) during follow-up, length of follow-up, major depressive disorder (MDD) subtypes at baseline and during follow-up and current vs. remitted MDD status at follow-up, severity during follow-up (number of symptoms of most severe major depressive episode (MDE), time spent in MDE, global assessment functioning (GAF) score, suicidality, hospitalization, psychotic features), relatives with MDD, number of different antidepressant compounds prior to baseline, and other antidepressants (Fluvoxamine, Bupropion, Reboxetine, Moclobémide, Trimipramine, Mianserin) during follow-up.

* *p* < 0.05, ** *p* < 0.01, *** *p* < 0.001.

Significant results are indicated in bold.

Table 5 displays the associations of use of AD classes and the specific compounds during follow-up with changes in adiposity markers by the period of AD use, adjusting for use of AD prior to baseline, occurrence of MDE prior to baseline, and during follow-up and depression severity. A steeper increase in BMI and waist circumference was observed in participants using any AD or any SSRI during the second period of the follow-up period, but not in those who used these drugs uniquely during the first period of the follow-up or during the entire follow-up. Among the SSRIs, fluoxetine and escitalopram prescribed during the second period of the follow-up were associated with a steeper increase in these two adiposity markers, whereas sertraline was only associated with a steeper increase in BMI. For fat mass, we observed a steeper increase in participants who used SSRIs during the entire follow-up and in those who used mirtazapine/trazodone exclusively during the first period of the follow-up. Regarding specific compounds, Fluoxetine prescribed during the second period of the follow-up as well as mirtazapine and paroxetine prescribed during the entire follow-up were associated with a steeper increase of fat mass.

**Table 5:** Change of adiposity markers during follow-up by timing of use of specific antidepressant during follow-up.

	Change in adiposity markers during follow-up						
	AD during first period of follow-up only		AD during second period of follow-up only		AD during 1st and 2^nd^ periods of follow-up		No AD
	β^a^	(95%CI)	β^a^	(95%CI)	β^a^	(95%CI)	β^a^
Body Mass Index [kg/m^2^] (*n* = 2462)							
Any antidepressants	0.24	(−0.12, 0.60)	0.39***	**(0.17, 0.60)**	0.18	(−0.16, 0.52)	0 (ref.)
TCA	0.25	(−0.53, 1.03)	0.42	(−0.08, 0.93)	−0.61	(−1.89, 0.67)	0 (ref.)
SSRI	0.16	(−0.28, 0.59)	0.46***	**(0.22, 0.69)**	0.14	(−0.34, 0.61)	
Mirtazapine/Trazodone	0.52	(−0.66, 1.70)	−0.18	(−0.68, 0.31)	−0.24	(−1.40, 0.92)	
Other^b^	0.53	(−0.42, 1.49)	0.13	(−0.27, 0.54)	0.18	(−0.59, 0.94)	
TCA							0 (ref.)
Clomipramine	−1.13	(−3.23, 0.97)	−0.13	(−1.16, 0.90)	—	—	
Amitriptyline	−0.25	(−1.70, 1.19)	0.41	(−0.35, 1.18)	−0.92	(−2.97, 1.12)	
Melitracen and psycholeptics	0.30	(−0.69, 1.28)	0.67	(−0.60, 1.95)	0.29	(−2.54, 3.11)	
SSRI							
Fluoxetine	0.68	(−0.01, 1.36)	0.49*	**(0.04, 0.95)**	−0.36	(−1.15, 0.42)	
Citalopram	−0.36	(−1.01, 0.30)	−0.18	(−0.60, 0.24)	0.35	(−0.76, 1.45)	
Paroxetine	−0.27	(−1.14, 0.60)	0.20	(−0.40, 0.79)	−0.17	(−1.71, 1.36)	
Sertraline	0.16	(−0.85, 1.17)	0.78*	**(0.15, 1.41)**	1.48	(−0.51, 3.48)	
Escitalopram	—	—	0.59***	**(0.26, 0.92)**	—	—	
Mirtazapine/Trazodone							
Mirtazapine	—	—	−0.43	(−1.09, 0.22)	0.30	(−0.66, 1.26)	
Trazodone	—	—	0.29	(−0.49, 1.07)	0.15	(−1.86, 2.15)	
SNRI							
Venlafaxine	0.47	(−0.50, 1.44)	0.05	(−0.51, 0.62)	0.28	(−0.54, 1.11)	
Duloxetine	—	—	0.20	(−0.33, 0.73)	—	—	
Waist circumference [cm] (*n* = 2475)							
Any antidepressants	0.38	(−1.15, 1.90)	1.47**	**(0.55, 2.39)**	1.28	(−0.18, 2.73)	0 (ref.)
TCA	2.78	(−0.57, 6.13)	1.97	(−0.14, 4.08)	3.90	(−1.58, 9.38)	0 (ref.)
SSRI	−0.15	(−2.01, 1.71)	1.33**	**(0.33, 2.33)**	0.98	(−1.06, 3.03)	
Mirtazapine/Trazodone	0.21	(−4.83, 5.26)	0.02	(−2.10, 2.14)	−2.09	(−7.08, 2.89)	
Other^b^	0.42	(−3.68, 4.53)	0.98	(−0.73, 2.70)	2.63	(−0.65, 5.92)	
TCA							0 (ref.)
Clomipramine	5.24	(−3.73, 14.21)	−1.25	(−5.39, 2.88)	—	—	
Amitriptyline	1.61	(−4.58, 7.80)	1.63	(−1.66, 4.91)	2.30	(−6.44, 11.04)	
Melitracen and psycholeptics	−0.98	(−5.19, 3.23)	2.35	(−3.10, 7.81)	6.62	(−5.45, 18.69)	
SSRI							
Fluoxetine	2.58	(−0.28, 5.43)	2.80**	**(0.86, 4.74)**	−0.71	(−4.04, 2.62)	
Citalopram	−0.63	(−3.42, 2.16)	−0.47	(−2.28, 1.34)	1.18	(−3.55, 5.91)	
Paroxetine	−1.50	(−5.20, 2.20)	−0.03	(−2.58, 2.53)	−3.37	(−9.93, 3.19)	
Sertraline	−0.24	(−4.55, 4.06)	1.51	(−1.12, 4.14)	4.24	(−4.28, 12.76)	
Escitalopram	—	—	1.97**	**(0.56, 3.37)**	—	—	
Mirtazapine/Trazodone							
Mirtazapine	—	—	−1.52	(−4.32, 1.29)	0.25	(−3.86, 4.35)	
Trazodone	—	—	2.07	(−1.25, 5.38)	−0.15	(−8.71, 8.42)	
SNRI							
Venlafaxine	0.41	(−3.74, 4.56)	0.05	(−2.37, 2.46)	3.15	(−0.37, 6.67)	
Duloxetine	—	—	1.14	(−1.11, 3.38)	—	—	
Fat mass [%] (*n* = 2079)							
Any antidepressants	0.34	(−0.73, 1.41)	−0.15	(−0.82, 0.51)	0.90	(−0.12, 1.91)	0 (ref.)
TCA	0.55	(−1.69, 2.79)	−1.13	(−2.58, 0.33)	−0.66	(−4.62, 3.30)	0 (ref.)
SSRI	0.55	(−0.73, 1.83)	0.61	(−0.12, 1.34)	1.83*	**(0.41, 3.26)**	
Mirtazapine/Trazodone	3.84*	**(0.28, 7.41)**	−1.04	(−2.58, 0.51)	1.48	(−2.41, 5.37)	
Other^b^	−2.17	(−4.82, 0.48)	−0.50	(−1.66, 0.66)	−0.89	(−3.08, 1.30)	
TCA							0 (ref.)
Clomipramine	−1.62	(−7.43, 4.19)	−0.45	(−3.29, 2.39)	—	—	
Amitriptyline	−0.12	(−4.12, 3.87)	−0.21	(−2.40, 1.99)	−0.98	(−6.63, 4.66)	
Melitracen and psycholeptics	1.35	(−1.52, 4.21)	−1.48	(−5.42, 2.47)	—	—	
SSRI							
Fluoxetine	1.58	(−0.42, 3.59)	1.55*	**(0.19, 2.92)**	1.92	(−0.40, 4.24)	
Citalopram	−0.28	(−2.22, 1.66)	−0.59	(−1.99, 0.80)	1.55	(−1.52, 4.61)	
Paroxetine	0.01	(−2.63, 2.64)	1.08	(−0.68, 2.84)	8.01*	**(0.13, 15.89)**	
Sertraline	0.73	(−2.04, 3.51)	1.84	(−0.08, 3.77)	1.30	(−4.17, 6.77)	
Escitalopram	—	—	0.91	(−0.12, 1.94)	−	−	
Mirtazapine/Trazodone							
Mirtazapine	—	—	−1.18	(−3.32, 0.96)	4.21**	**(1.21, 7.21)**	
Trazodone	—	—	−0.65	(−2.97, 1.68)	−0.95	(−6.46, 4.55)	
SNRI							
Venlafaxine	−2.08	(−4.76, 0.61)	0.19	(−1.48, 1.85)	−0.58	(−2.94, 1.78)	
Duloxetine	—	—	−0.70	(−2.19, 0.80)	—	—	

Models assessing the association between change in adiposity markers and specific compounds of AD were additionally adjusted for other antidepressants (Fluvoxamine, Bupropion, Reboxetine, Moclobémide, Trimipramine, Mianserin) during follow-up.

*AD* antidepressant, *TCA* Tricyclic antidepressants, *SSRI* Selective Serotonin Reuptake Inhibitors, *95%CI* 95% confidence interval. *ref*. reference group.

^a^Adjusted for socio-demographic characteristics (sex, age, socio-economic status, living alone during follow-up), early physical and sexual abuse, adiposity marker levels at baseline, behavioral factors (physical inactivity, smoking status, number of drinks per week) during follow-up, anxiety disorders and illicit drug dependence during follow-up, possibly weight gain inducing medication (other than antidepressants) during follow-up, length of follow-up, major depressive disorder (MDD) subtypes at baseline and during follow-up and current vs. remitted MDD status at follow-up, severity during follow-up (number of symptoms of most severe major depressive episode (MDE), time spent in MDE, global assessment functioning (GAF) score, suicidality, hospitalization, psychotic features), relatives with MDD, and number of different antidepressant compounds prior to baseline.

^b^SNRI (Serotonin-Noradrenaline Reuptake Inhibitors)/NDRI (Noradrenaline Reuptake Inhibitors)/MAOI(Monoamine Oxidase Inhibitors).

* *p* < 0.05, ** *p* < 0.01, *** *p* < 0.001

Significant results are indicated in bold.

### Adjustment using propensity scores

The results of the models with adjustment using propensity scores are presented in Tables S3–S6*in the Supplement*. These complementary analyses provided very similar results to those of Model 3. Only 7 results were no longer significant or became significant after adjustment with the propensity score. These changes involved: (1) the number of AD compounds used prior to baseline shortly failed to remain significantly associated with change in waist circumference (β (95%CI) = −0.25 (−0.53, 0.02), *p* = 0.073, Table S3); (2) TCA use during the follow-up became marginally associated with an increase in BMI during the follow-up (β (95%CI) = 0.43 (0.00, 0.87), *p* = 0.049, Table S4); (3) SSRI use prior to baseline shortly failed to reach significance for change in waist circumference (β (95%CI) = −0.85 (−1.73, 0.04), *p* = 0.061, Table S4); (4) Mirtazapine use during the second period of the follow-up became significantly associated with change in BMI (β (95%CI) = −0.72 (−1.37, −0.07), *p* = 0.030, Table S6); (5) Mirtazapine use during the first period of the follow-up and change in fat mass failed to reach the level of statistical significance (β (95%CI) = 3.62 (−0.15, 7.38), *p* = 0.060, Table S6); (6) Paroxetine use during the first and the second periods of the follow-up and change in fat mass failed to reach the level of statistical significance (β (95%CI) = 6.84 (−1.39, 15.07), *p* = 0.103, Table S6); and (7) Venlafaxine use during the first period of the follow-up became significantly associated with change in fat mass (β (95%CI) = −3.10 (−5.94, −0.27), *p* = 0.032, Table S6).

### Analyses with a 5% adiposity marker increase in participants as the outcome variables

These additional analyses confirmed that most of the significant associations between AD use and adiposity marker increase during the follow-up were paralleled by higher proportions of participants with at least a 5% adiposity marker increase among AD users. Indeed compared to non-users, AD users during the follow-up revealed at least 5% increase in BMI during follow-up (41.7% vs. 29.3%; OR (95%CI) = 1.67 (1.27, 2.19), *p* < .001) and at least 5% increase in waist circumference (53.2% vs. 40.9%; OR (95%CI) = 1.51 (1.16, 1.98), *p* = 0.003) more frequently according to the fully adjusted models (Table S7). With respect to AD classes, SSRI users during the follow-up were more likely to have at least a 5% increase in BMI (43.3% vs. 29.3%; OR (95%CI) = 1.63 (1.18, 2.25), *p* = 0.003) and at least 5% increase in waist circumference (53.4% vs. 40.9%; OR (95%CI) = 1.42 (1.03, 1.96), *p* = 0.030), whereas TCA use was only associated with an elevated frequency of at least 5% increase in waist circumference during the follow-up (69.8% vs. 40.9%; OR (95%CI) = 3.22 (1.66, 6.27), *p* < .001, Table S8). Among the specific compounds, escitalopram use during the follow-up was also associated with a higher likelihood of at least 5% increase in BMI (51.7% vs. 29.3%; OR (95%CI) = 2.31 (1.43, 3.72), *p* < .001) and at least 5% increase in waist circumference (59.8% vs. 40.9; OR (95%CI) = 1.79 (1.10, 2.91), *p* = 0.019), whereas fluoxetine and sertraline use during the follow-up, which were both associated with a steeper increase in some of the adiposity markers, were not associated with a higher likelihood of at least a 5% increase in adiposity markers during the follow-up (Table S9). In contrast, amitriptyline use during the follow-up, which was not associated with a steeper increase in adiposity markers, revealed an association with a higher likelihood of at least a 5% increase in waist circumference (70.0% vs. 40.9%; OR (95%CI) = 3.08 (1.12, 8.49), *p* = 0.030, Table S9).

## Discussion

The 5.5-year follow-up of a cohort recruited from the general population allowed us to jointly assess the associations of the use of ADs prior to baseline and the use of ADs during this follow-up with changes in measured BMI, waist circumference, and fat mass, accounting for a series of potential confounders. The most salient findings were: (1) independently of the effects of depressive episodes and depression severity as well as the propensity adjustment approach, the number of ADs, mainly SSRIs and TCAs, prescribed prior to the baseline was associated with a lower increase in BMI and waist circumference, whereas the use of ADs during the follow-up was associated with a higher increase in BMI and waist circumference during the follow-up; (2) within the class of SSRIs, the use of fluoxetine, sertraline or escitalopram during follow-up was associated with a steeper increase in adiposity markers; (3) the associations between SSRIs with BMI and waist circumference were only observed when the SSRIs were used during the second but not the first period of follow-up; and (4) there was no evidence for differential associations between AD use and changes in adiposity markers between men and women.

The higher increase in BMI among AD users during follow-up also involved a more than 60% elevated risk of at least a 5% increase in BMI, which is usually considered as clinically relevant, and a more than 50% elevated risk of at least a 5% increase in waist circumference as compared to those who did not use these drugs. Within AD classes, SSRI users had a more than 60% and 40% elevated risks of at least a 5% increase in BMI and waist circumference, respectively, whereas TCA users were at an even more than tripled risk of at least a 5% increase in waist circumference. Our observation of a steeper increase in BMI during TCA and SSRI use during follow-up is compatible with previous research that documented elevated weight gain in people using ADs over periods of over 1 year [12–14]. Although we only observed an association with waist circumference for any TCA use, this finding is in line with those of clinical and observational studies that constantly revealed associations between this AD class and weight gain [9, 35, 36]. Moreover, extending previous evidence, we could demonstrate that a sustained use of SSRIs is not only associated with a steeper increase in BMI [7, 10, 12, 14, 37, 38], but also a steeper increase in waist circumference (fluoxetine, escitalopram) and fat mass (fluoxetine). However, the results with respect to fluoxetine need to be interpreted with caution. Indeed, despite the observed steeper increase in all adiposity markers among the users of this compound, it was not associated with a higher likelihood of at least a 5% increase in any of these markers. Hence, the clinical relevance of the observed steeper increase in adiposity markers associated with this compound remains doubtful.

In addition to previous cohort studies, which usually did not separately assess the effects of drug treatment prior to baseline and during follow-up, as well as changes of drug prescriptions during follow-up, we could show that (1) the number of drug treatments prior to baseline was associated with a lower increase in BMI (TCA, SSRI) and waist circumference (SSRI) during follow-up, and (2) people treated with SSRIs uniquely during the first period of the follow-up did not reveal a steeper increase in BMI and waist circumference than those without such treatment. These two observations would be compatible with an increase in adiposity markers during the period of treatment with the assessed ADs restricted to the treatment period, followed by a normalization of these markers after the cessation of treatment. The somewhat counterintuitive finding that AD treatment during the whole follow-up was not associated with the size of increase in BMI and waist circumference might indicate ceiling effects. Indeed, participants with treatment throughout the whole follow-up were likely to have already used these ADs for many years prior to baseline, and weight gain associated with the use of these drugs may have ceased before study intake. With respect to fat mass, we found no evidence for a ceiling effect. Indeed, those who had used any SSRI or mirtazapine during the whole follow-up had a significantly steeper increase in this adiposity marker. The observed association between mirtazapine and cardiometabolic markers corroborates findings of previous research [7, 10, 39–41]. Longer follow-up studies with intermediate measures would be necessary to assess the timing of potential ceiling effects in the increase of cardiometabolic markers among those who are under long-term treatment with ADs. Our results were adjusted for multiple potential confounder variables including sociodemographic characteristics, health-related behaviors, the presence of non-mood disorders, and other medication (Model 1–3). Interestingly, all the negative associations between AD use prior to baseline and adiposity marker changes reached the level of statistical significance only after adjustments in the models, which suggested that the absence of significant negative associations in non-adjusted analyses may have been blurred by confounding. Mediation would be an alternative explanation, assuming that several of these variables, particularly behaviors (1) were affected by AD use, (2) persisted after cessation of treatment, and (3) favored adiposity marker changes during follow-up. Under this assumption, the emergence of negative associations in adjusted analyses would have been the result of the compensation of a decrease in adiposity markers after cessation of AD treatment (as evidenced in adjusted analyses) through persisting treatment-related behaviors predisposing to adiposity (e.g., elevated food intake or lack of exercising). In contrast to most previous research, which could not statistically disentangle between the effects of drugs and the depression itself, a second set of analyses were additionally adjusted for the effects of depressive episodes (Model 2) and their severity (Model 3), still supporting an association between AD use during follow-up and an elevated increase in adiposity markers. Moreover, the adjustment for adiposity marker levels at baseline should have further mitigated the risk of indication bias, if a pre-existing tendency towards adiposity had motivated health providers to more frequently prescribe compounds reputed for not inducing weight gain, e.g., SSRIs, in these participants.

In contrast to two previous studies [21, 22], which suggested higher weight gain in women using ADs than men, our results did not provide evidence for such sex-specific differences. However, given large methodological differences across these studies results are difficult to compare. Indeed, one of the two previous studies used a clinical sample and self-reported weight data [21], whereas the other included a 20 year older cohort from the community [22].

Several mechanistic hypotheses have been posed regarding the potential weight-inducing effects of sustained AD use. TCAs enfold a very wide and non-selective activity against different molecular targets, which comprises inhibition of serotonin, noradrenaline, and dopamine reuptake, and antagonistic effects against histamine H1 and muscarinic acetylcholine receptors [42]. Antihistaminic and anticholinergic effects have been associated with increased carbohydrate craving and increased appetite [43]. Likewise, prolonged inhibition of serotonin reuptake has been associated with carbohydrate craving, while acute binding to serotonin reuptake receptors such as 5-HT2C by SSRIs such as fluoxetine has been shown to reduce appetite [44, 45]. This may explain the partially heterogeneous effects on weight seen with SSRIs dependent on the length of the observational time frame and treatment.

Our results need to be viewed in the light of several limitations. First, given that our data are observational, our results could be affected by confounding effects such as the physician’s selection of a specific AD compound (e.g., fluoxetine) in consideration of potential weight gain. However, by adjusting our analyses for multiple depression severity markers we have minimized the risk of indication bias due to more frequent prescription of ADs in more severe forms of MDD, which may be associated with elevated weight gain. Second, our treatment data mainly relied on self-reports. Inaccurate reporting could have induced non-differential bias resulting in an underestimation of the size of the measured associations. Third, additional bias could have been introduced by the non-participation of 23.2% of the initial cohort. Fourth, the number of treated participants is low for specific compounds in some of the given periods, which involves the risk of false negative findings.

## Conclusion

The results of the study are compatible with an increase in adiposity markers related to sustained treatment with TCAs, SSRIs, and mirtazapine, which however may return to normal levels after cessation of treatment. The long-term administration of these AD classes may be necessary for attenuating the symptoms of chronic depressive episodes or preventing recurrent episodes, which bear the risk of high individual suffering, suicidality as well as impairment of professional and social life. However, these benefits should also be carefully weighed against the risk of weight gain and its potential consequences.

### Supplementary information

Supplementary information

## Data Availability

The data of CoLaus|PsyCoLaus study used in this article cannot be fully shared as they contain potentially sensitive personal information on participants. According to the Ethics Committee for Research of the Canton of Vaud, sharing these data would be a violation of the Swiss legislation with respect to privacy protection. However, coded individual-level data that do not allow researchers to identify participants are available upon request to researchers who meet the criteria for data sharing of the CoLaus|PsyCoLaus Datacenter (CHUV, Lausanne, Switzerland). Any researcher affiliated to a public or private research institution who complies with the CoLaus|PsyCoLaus standards can submit a research application to research.colaus@chuv.ch or research.psycolaus@chuv.ch. Proposals requiring baseline data only will be evaluated by the baseline (local) Scientific Committee (SC) of the CoLaus and PsyCoLaus studies. Proposals requiring follow-up data will be evaluated by the follow-up (multicentric) SC of the CoLaus|PsyCoLaus cohort study. Detailed instructions for gaining access to the CoLaus|PsyCoLaus data used in this study are available at www.colaus-psycolaus.ch/professionals/how-to-collaborate/.
