# Supplementary material for: Long-term changes in adiposity markers during and after antidepressant therapy in a community cohort
Source: Transl Psychiatry. 2024 Aug 13;14:330. doi: 10.1038/s41398-024-03032-5 (PMC11322521; doi:10.1038/s41398-024-03032-5)

**Supplementary information**

**Long-term changes in adiposity markers during and after antidepressant therapy in a community cohort**

Jessica Mwinyi^*^, MD, PhD, Marie-Pierre F. Strippoli^*^, MSc, Sofia H. Kanders, PhD, Helgi B. Schiöth, PhD, Chin B. Eap, PhD, Aurélie M. Lasserre, MD, PhD, Pedro Marques-Vidal, MD, PhD, Caroline L. Vandeleur, PhD, Martin Preisig, MD, MPH

**Table S1: Definition and distribution of groups of antidepressants (n=2479)**

| **Group** | **Name** | **ATC code** | **No. (%)** |
| --- | --- | --- | --- |
| TCA | Imipramine | N06AA02 | 0 (0.0) |
|  | Clomipramine | N06AA04 | 11 (0.4) |
|  | Trimipramine | N06AA06 | 7 (0.3) |
|  | Dibenzepin | N06AA08 | 0 (0.0) |
|  | Amitriptyline | N06AA09 | 20 (0.8) |
|  | Maprotiline | N06AA21 | 0 (0.0) |
|  | Mianserin | N06AX03 | 3 (0.1) |
|  | Melitracen and psycholeptics | N06CA02 | 16 (0.6) |
| SSRI | Fluoxetine | N06AB03 | 76 (3.1) |
|  | Citalopram | N06AB04 | 77 (3.1) |
|  | Paroxetin | N06AB05 | 40 (1.6) |
|  | Sertraline | N06AB06 | 32 (1.3) |
|  | Fluvoxamine | N06AB08 | 3 (0.1) |
|  | Escitalopram | N06AB10 | 87 (3.5) |
| SNRI | Venlafaxine | N06AX16 | 50 (2.0) |
|  | Duloxetine | N06AX21 | 33 (1.3) |
| NDRI | Bupropion | N06AX12 | 2 (0.1) |
|  | Reboxetine | N06AX18 | 1 (0.0) |
| MOAI | Moclobémide | N06AG02 | 2 (0.1) |
| Tra-Mir | Trazodone | N06AX05 | 16 (0.6) |
|  | Mirtazapine | N06AX11 | 31 (1.3) |

ATC : anatomical therapeutic chemical ; TCA : tricyclic antidepressants, SSRI : selective serotonin reuptake inhibitors, SNRI : serotonin-noradrenaline reuptake inhibitors, NDRI : noradrenaline reuptake inhibitors, MAOI : Monoamine oxidase inhibitors, Tra-Mir : trazodone or mirtazapine

**Table S2: Drugs possibly increasing weight taken into account in the statistical analysis**

| Aldesleukin | Amisulpride | Aripiprazole |  |
| --- | --- | --- | --- |
| Carvedilol | Chlorpromazine | Chlorprothixene |  |
| Clobazam | Clomifene | Clozapine |  |
| Danazol | Desogestrel | Dexamethasone |  |
| Doxepin | Drospirenone (+combination) | Estradiol (+combination) |  |
| Ethinylestradiol (+combination) | Etonogestrel | Etoricoxib |  |
| Flupentixol | Gabapentin | Glatiramer |  |
| Insulin | Ketazolam | Ketotifen |  |
| Levocetirizine | Levonorgestrel | Lithium |  |
| Megestrol | Metformin (+combination) | Minoxidil |  |
| Olanzapine | Paliperidone | Perphenazine |  |
| Pioglitazone | Pregabalin | Progesterone |  |
| Quetiapine | Risperidone | Rosiglitazone (+combination) |  |
| Sertindole | Sulpiride | Terazosin |  |
| Tibolone | Toremifene | Valproate |  |
| Vigabatrin | Zuclopenthixol |  |  |

The list was extracted from (http://www.micromedex.com)^1^ and Compendium Suisse de Médicaments®.^2^ Bâle (Suisse): Documed S.A, 2010.

1.http://www.micromedex.com. (Accessed November 15, 2011)

2.Compendium Suisse de Médicaments®. Bâle (Suisse): Documed S.A.; 2010.

**Table S3: Change of adiposity markers during follow-up by any antidepressant use prior to baseline and during follow-up with adjustment for the propensity score**

|  | **Change in adiposity markers** | |
| --- | --- | --- |
|  | **β^1^** | **(95%CI)** |
| **Body Mass Index [kg/m^2^] (n=2462)** |  |  |
| Antidepressants prior to baseline |  |  |
| Number of compounds | **-0.11***** | **(-0.17,-0.05)** |
| Antidepressants during follow-up |  |  |
| Any antidepressants | **0.36***** | **(0.17,0.54)** |
| No antidepressants (ref.) | 0 (ref.) | - |
| **Waist circumference [cm] (n=2475)** |  |  |
| Antidepressants prior to baseline |  |  |
| Number of compounds | -0.25 | (-0.53,0.02) |
| Antidepressants during follow-up |  |  |
| Any antidepressants | **1.26**** | **(0.48,2.04)** |
| No antidepressants (ref.) | 0 (ref.) | - |
| **Fat mass [%] (n=2079)** |  |  |
| Antidepressants prior to baseline |  |  |
| Number of compounds | -0.11 | (-0.31,0.09) |
| Antidepressants during follow-up |  |  |
| Any antidepressants | 0.01 | (-0.57,0.60) |
| No antidepressants (ref.) | 0 (ref.) | - |

95%CI: 95% confidence interval. ref.: reference group. *p<0.05, **p<0.01, ***p<0.001.

^1^ Adjusted for the propensity score, and adiposity marker levels at baseline.

*Propensity score: probability to report lifetime antidepressants use.*

*Confounders used to compute the propensity score: socio-demographic characteristics (sex, age, socio-economic status, living alone during follow-up), early physical and sexual abuse, behavioral factors (physical inactivity, smoking status, number of alcohol drinks per week) during follow-up, anxiety disorders and illicit drug dependence during follow-up, possibly weight gain inducing medication (other than antidepressants) during follow-up, and length of follow-up, major depressive disorder (MDD) subtypes at baseline and during follow-up and current vs. remitted MDD status at follow-up, severity during follow-up (number of symptoms of most severe major depressive episode (MDE), time spent in MDE, global assessment functioning (GAF) score, suicidality, hospitalization, psychotic features), and relatives with MDD.*

**Table S4: Change of adiposity markers during follow-up by use of antidepressant class prior to baseline and during follow-up with adjustment for the propensity score**

|  | **Change in adiposity markers** | |
| --- | --- | --- |
|  | **β^1^** | **(95%CI)** |
| **Body Mass Index [kg/m^2^] (n=2462)** |  |  |
| Antidepressants prior to baseline |  |  |
| TCA | **-0.55**** | **(-0.91,-0.18)** |
| SSRI | **-0.24*** | **(-0.45,-0.03)** |
| Mirtazapine/Trazodone | -0.12 | (-0.72,0.48) |
| Other^2^ | -0.15 | (-0.64,0.33) |
| No antidepressants (ref.) | 0 (ref.) | - |
| Antidepressants during follow-up |  |  |
| TCA | **0.43*** | **(0.00,0.87)** |
| SSRI | **0.38***** | **(0.17,0.60)** |
| Mirtazapine/ Trazodone | -0.13 | (-0.56,0.31) |
| Other^2^ | 0.17 | (-0.19,0.53) |
| No antidepressants (ref.) | 0 (ref.) | - |
| **Waist circumference [cm] (n=2475)** |  |  |
| Antidepressants prior to baseline |  |  |
| TCA | -0.62 | (-2.15,0.91) |
| SSRI | -0.85 | (-1.73,0.04) |
| Mirtazapine/Trazodone | -1.33 | (-3.88,1.21) |
| Other^2^ | 0.05 | (-2.01,2.12) |
| No antidepressants (ref.) | 0 (ref.) | - |
| Antidepressants during follow-up |  |  |
| TCA | **2.38*** | **(0.57,4.19)** |
| SSRI | **1.16*** | **(0.24,2.07)** |
| Mirtazapine/ Trazodone | -0.36 | (-2.20,1.49) |
| Other^2^ | 0.67 | (-0.85,2.20) |
| No antidepressants (ref.) | 0 (ref.) | - |
| **Fat mass [%] (n=2079)** |  |  |
| Antidepressants prior to baseline |  |  |
| TCA | -0.23 | (-1.38,0.91) |
| SSRI | 0.08 | (-0.58,0.73) |
| Mirtazapine/Trazodone | -0.51 | (-2.51,1.48) |
| Other^2^ | -0.58 | (-2.07,0.91) |
| No antidepressants (ref.) | 0 (ref.) | - |
| Antidepressants during follow-up |  |  |
| TCA | -0.12 | (-1.45,1.20) |
| SSRI | 0.51 | (-0.18,1.20) |
| Mirtazapine/Trazodone | -0.42 | (-1.84,1.00) |
| Other^2^ | -0.87 | (-1.96,0.21) |
| No antidepressants (ref.) | 0 (ref.) | - |

TCA: Tricyclic antidepressants; SSRI: Selective Serotonin Reuptake Inhibitors; 95%CI: 95% confidence interval. ref.: reference group. *p<0.05, **p<0.01, ***p<0.001.

^1^ Adjusted for the propensity score, and adiposity marker levels at baseline.

^2^ SNRI (Serotonin-Noradrenaline Reuptake Inhibitors)/NDRI (Noradrenaline Reuptake Inhibitors)/MAOI(Monoamine Oxidase Inhibitors).

*Propensity score: probability to report lifetime antidepressants use.*

*Confounders used to compute the propensity score: socio-demographic characteristics (sex, age, socio-economic status, living alone during follow-up), early physical and sexual abuse, behavioral factors (physical inactivity, smoking status, number of alcohol drinks per week) during follow-up, anxiety disorders and illicit drug dependence during follow-up, possibly weight gain inducing medication (other than antidepressants) during follow-up, and length of follow-up, major depressive disorder (MDD) subtypes at baseline and during follow-up and current vs. remitted MDD status at follow-up, severity during follow-up (number of symptoms of most severe major depressive episode (MDE), time spent in MDE, global assessment functioning (GAF) score, suicidality, hospitalization, psychotic features), and relatives with MDD.*

**Table S5: Change of adiposity markers during follow-up by use of specific antidepressants during follow-up with adjustment for the propensity score**

|  | **Change in adiposity markers during follow-up** | | | | | |
| --- | --- | --- | --- | --- | --- | --- |
|  | **Body Mass Index [kg/m^2^]**  **(n=2462)** | | **Waist circumference [cm]**  **(n=2475)** | | **Fat mass [%]**  **(n=2079)** | |
|  | **β^1^** | **(95%CI)** | **β^1^** | **(95%CI)** | **β^1^** | **(95%CI)** |
| TCA |  |  |  |  |  |  |
| Clomipramine | -0.33 | (-1.26,0.59) | 0.54 | (-3.20,4.28) | -0.12 | (-2.81,2.58) |
| Amitriptyline | 0.23 | (-0.41,0.87) | 1.76 | (-0.97,4.48) | -0.71 | (-2.62,1.20) |
| Melitracen and psycholeptics | 0.45 | (-0.30,1.20) | 1.42 | (-1.74,4.58) | 0.85 | (-1.58,3.28) |
| SSRI |  |  |  |  |  |  |
| Fluoxetine | **0.41*** | **(0.06,0.76)** | **2.19**** | **(0.72,3.66)** | **1.37*** | **(0.27,2.46)** |
| Citalopram | -0.17 | (-0.51,0.17) | -0.45 | (-1.90,1.00) | 0.02 | (-1.11,1.14) |
| Paroxetine | -0.06 | (-0.53,0.41) | -0.84 | (-2.81,1.14) | 0.80 | (-0.71,2.30) |
| Sertraline | **0.68*** | **(0.16,1.20)** | 1.40 | (-0.77,3.58) | 1.09 | (-0.53,2.71) |
| Escitalopram | **0.58***** | **(0.26,0.90)** | **1.85**** | **(0.48,3.21)** | 0.04 | (-1.02,1.09) |
| Mirtazapine/Trazodone |  |  |  |  |  |  |
| Mirtazapine | -0.38 | (-0.91,0.15) | -1.53 | (-3.79,0.72) | -0.07 | (-1.90,1.75) |
| Trazodone | 0.56 | (-0.16,1.29) | 1.76 | (-1.32,4.83) | -1.16 | (-3.41,1.09) |
| SNRI |  |  |  |  |  |  |
| Venlafaxine | 0.22 | (-0.21,0.64) | 0.76 | (-1.04,2.56) | -1.06 | (-2.34,0.23) |
| Duloxetine | 0.10 | (-0.41,0.62) | 0.28 | (-1.89,2.45) | -0.50 | (-2.04,1.03) |
| No antidepressants (ref.) | 0 (ref.) | - | 0 (ref.) | - | 0 (ref.) | - |

TCA: tricyclic antidepressants; SSRI: selective serotonin reuptake inhibitors; SNRI: serotonin-noradrenaline reuptake inhibitors; 95%CI: 95% confidence interval. ref.: reference group. *p<0.05, **p<0.01, ***p<0.001.

^1^ Adjusted for the propensity score, adiposity marker levels at baseline, number of different antidepressant compounds prior to baseline, and other antidepressants (Fluvoxamine, Bupropion, Reboxetine, Moclobémide, Trimipramine, Mianserin) during follow-up.

*Propensity score: probability to report lifetime antidepressants use.*

*Confounders used to compute the propensity score: socio-demographic characteristics (sex, age, socio-economic status, living alone during follow-up), early physical and sexual abuse, behavioral factors (physical inactivity, smoking status, number of alcohol drinks per week) during follow-up, anxiety disorders and illicit drug dependence during follow-up, possibly weight gain inducing medication (other than antidepressants) during follow-up, and length of follow-up, major depressive disorder (MDD) subtypes at baseline and during follow-up and current vs. remitted MDD status at follow-up, severity during follow-up (number of symptoms of most severe major depressive episode (MDE), time spent in MDE, global assessment functioning (GAF) score, suicidality, hospitalization, psychotic features), and relatives with MDD.*

**Table S6: Change of adiposity markers during follow-up by timing of use of specific antidepressant during follow-up with adjustment for the propensity score**

|  | **Change in adiposity markers during follow-up** | | | | | | |
| --- | --- | --- | --- | --- | --- | --- | --- |
|  | **AD during first period**  **of follow-up only** | | **AD during second period**  **of follow-up only** | | **AD during 1st and 2nd**  **periods of follow-up** | | **No AD** |
|  | **β^1^** | **(95%CI)** | **β^1^** | **(95%CI)** | **β^1^** | **(95%CI)** | **β^1^** |
| **Body Mass Index [kg/m^2^] (n=2462)** |  |  |  |  |  |  |  |
| Any antidepressants | 0.33 | (-0.03,0.68) | **0.41***** | **(0.20,0.62)** | 0.22 | (-0.12,0.56) | 0 (ref.) |
| TCA | 0.34 | (-0.44,1.13) | 0.41 | (-0.09,0.92) | -0.36 | (-1.64,0.92) | 0 (ref.) |
| SSRI | 0.21 | (-0.23,0.65) | **0.49***** | **(0.26,0.72)** | 0.12 | (-0.36,0.60) |  |
| Mirtazapine/Trazodone | 0.68 | (-0.51,1.87) | -0.30 | (-0.79,0.19) | -0.08 | (-1.25,1.08) |  |
| Other^2^ | 0.60 | (-0.37,1.56) | 0.08 | (-0.31,0.47) | 0.20 | (-0.57,0.97) |  |
| TCA |  |  |  |  |  |  | 0 (ref.) |
| Clomipramine | -0.70 | (-2.81,1.42) | -0.18 | (-1.22,0.87) | - | - |  |
| Amitriptyline | -0.05 | (-1.51,1.41) | 0.52 | (-0.26,1.29) | -0.74 | (-2.78,1.30) |  |
| Melitracen and psycholeptics | 0.36 | (-0.62,1.35) | 0.69 | (-0.60,1.98) | -0.06 | (-2.91,2.79) |  |
| SSRI |  |  |  |  |  |  |  |
| Fluoxetine | 0.66 | (-0.03,1.35) | **0.50*** | **(0.04,0.96)** | -0.28 | (-1.06,0.50) |  |
| Citalopram | -0.35 | (-1.01,0.30) | -0.19 | (-0.61,0.23) | 0.24 | (-0.85,1.33) |  |
| Paroxetine | -0.19 | (-1.06,0.68) | 0.09 | (-0.50,0.69) | -0.33 | (-1.85,1.18) |  |
| Sertraline | 0.18 | (-0.83,1.20) | **0.85**** | **(0.22,1.48)** | 1.54 | (-0.48,3.55) |  |
| Escitalopram | - | - | **0.61***** | **(0.29,0.94)** | - | - |  |
| Mirtazapine/Trazodone |  |  |  |  |  |  |  |
| Mirtazapine | - | - | **-0.72*** | **(-1.37,-0.07)** | 0.44 | (-0.53,1.41) |  |
| Trazodone | - | - | 0.49 | (-0.30,1.27) | 0.44 | (-1.58,2.45) |  |
| SNRI |  |  |  |  |  |  |  |
| Venlafaxine | 0.53 | (-0.45,1.50) | 0.00 | (-0.56,0.56) | 0.33 | (-0.50,1.16) |  |
| Duloxetine | - | - | 0.10 | (-0.42,0.62) | - | - |  |
| **Waist circumference [cm] (n=2475)** |  |  |  |  |  |  |  |
| Any antidepressants | 0.44 | (-1.07,1.96) | **1.49**** | **(0.59,2.39)** | 1.25 | (-0.19,2.70) | 0 (ref.) |
| TCA | 2.89 | (-0.45,6.23) | 2.08 | (-0.03,4.20) | 2.78 | (-2.66,8.22) | 0 (ref.) |
| SSRI | -0.13 | (-1.99,1.73) | **1.42**** | **(0.43,2.40)** | 0.83 | (-1.21,2.87) |  |
| Mirtazapine/Trazodone | 0.72 | (-4.33,5.78) | -0.48 | (-2.58,1.62) | -1.04 | (-6.00,3.92) |  |
| Other^2^ | 0.16 | (-3.95,4.27) | 0.63 | (-1.03,2.30) | 2.27 | (-1.02,5.56) |  |
| TCA |  |  |  |  |  |  | 0 (ref.) |
| Clomipramine | 6.77 | (-2.21,15.75) | -1.11 | (-5.26,3.04) | - | - |  |
| Amitriptyline | 1.65 | (-4.54,7.84) | 1.72 | (-1.55,5.00) | 2.49 | (-6.15,11.14) |  |
| Melitracen and psycholeptics | -1.21 | (-5.39,2.96) | 2.49 | (-2.98,7.96) | 4.95 | (-7.14,17.04) |  |
| SSRI |  |  |  |  |  |  |  |
| Fluoxetine | 2.36 | (-0.49,5.20) | **3.26**** | **(1.32,5.20)** | -0.16 | (-3.47,3.15) |  |
| Citalopram | -0.65 | (-3.44,2.13) | -0.56 | (-2.34,1.22) | 0.02 | (-4.60,4.63) |  |
| Paroxetine | -0.84 | (-4.54,2.85) | -0.59 | (-3.12,1.94) | -3.75 | (-10.18,2.68) |  |
| Sertraline | -0.45 | (-4.75,3.85) | 1.95 | (-0.67,4.57) | 3.47 | (-5.08,12.03) |  |
| Escitalopram | - | - | **1.89**** | **(0.50,3.28)** | - | - |  |
| Mirtazapine/Trazodone |  |  |  |  |  |  |  |
| Mirtazapine | - | - | -2.37 | (-5.13,0.38) | 0.81 | (-3.30,4.91) |  |
| Trazodone | - | - | 2.03 | (-1.28,5.35) | 1.12 | (-7.43,9.67) |  |
| SNRI |  |  |  |  |  |  |  |
| Venlafaxine | 0.27 | (-3.88,4.42) | 0.09 | (-2.29,2.46) | 2.86 | (-0.66,6.38) |  |
| Duloxetine | - | - | 0.56 | (-1.64,2.76) | - | - |  |
| **Fat mass [%] (n=2079)** |  |  |  |  |  |  |  |
| Any antidepressants | 0.50 | (-0.61,1.62) | -0.32 | (-1.00,0.37) | 0.42 | (-0.65,1.48) | 0 (ref.) |
| TCA | 1.10 | (-1.26,3.46) | -0.71 | (-2.24,0.83) | -0.86 | (-4.98,3.26) | 0 (ref.) |
| SSRI | 0.60 | (-0.75,1.95) | 0.48 | (-0.28,1.23) | **1.59*** | **(0.09,3.09)** |  |
| Mirtazapine/Trazodone | 3.62 | (-0.15,7.38) | -1.58 | (-3.20,0.03) | 2.76 | (-1.34,6.85) |  |
| Other^2^ | **-3.10*** | **(-5.88,-0.31)** | -0.64 | (-1.82,0.55) | -1.50 | (-3.81,0.82) |  |
| TCA |  |  |  |  |  |  | 0 (ref.) |
| Clomipramine | 0.49 | (-5.66,6.64) | 0.10 | (-2.92,3.11) | - | - |  |
| Amitriptyline | -0.30 | (-4.54,3.95) | -0.49 | (-2.80,1.82) | -1.84 | (-7.72,4.04) |  |
| Melitracen and psycholeptics | 1.20 | (-1.81,4.21) | -0.37 | (-4.56,3.82) | - | - |  |
| SSRI |  |  |  |  |  |  |  |
| Fluoxetine | 1.15 | (-0.96,3.25) | **1.56*** | **(0.11,3.00)** | 1.60 | (-0.84,4.03) |  |
| Citalopram | 0.06 | (-1.99,2.11) | -0.21 | (-1.66,1.24) | 1.88 | (-1.26,5.03) |  |
| Paroxetine | 0.88 | (-1.91,3.67) | 0.67 | (-1.18,2.51) | 6.84 | (-1.39,15.07) |  |
| Sertraline | 0.02 | (-2.91,2.95) | 1.94 | (-0.09,3.97) | 0.61 | (-5.20,6.43) |  |
| Escitalopram | - | - | 0.26 | (-0.81,1.33) | - | - |  |
| Mirtazapine/Trazodone |  |  |  |  |  |  |  |
| Mirtazapine | - | - | -1.59 | (-3.83,0.65) | **4.45**** | **(1.27,7.63)** |  |
| Trazodone | - | - | -1.79 | (-4.24,0.66) | 0.62 | (-5.19,6.43) |  |
| SNRI |  |  |  |  |  |  |  |
| Venlafaxine | **-3.10*** | **(-5.94,-0.27)** | -0.25 | (-1.98,1.48) | -1.32 | (-3.81,1.18) |  |
| Duloxetine | - | - | -0.59 | (-2.14,0.97) | - | - |  |

AD: antidepressant; TCA: Tricyclic antidepressants; SSRI: Selective Serotonin Reuptake Inhibitors; 95%CI: 95% confidence interval. ref.: reference group. *p<0.05, **p<0.01, ***p<0.001

^1^ Adjusted for the propensity score, adiposity marker levels at baseline, and number of different antidepressant compounds prior to baseline.

Models assessing the association between change in adiposity markers and specific compounds of AD were additionally adjusted for other antidepressants (Fluvoxamine, Bupropion, Reboxetine, Moclobémide, Trimipramine, Mianserin) during follow-up.

^2^ SNRI (Serotonin-Noradrenaline Reuptake Inhibitors)/NDRI (Noradrenaline Reuptake Inhibitors)/MAOI(Monoamine Oxidase Inhibitors).

*Propensity score: probability to report lifetime antidepressants use.*

*Confounders used to compute the propensity score: socio-demographic characteristics (sex, age, socio-economic status, living alone during follow-up), early physical and sexual abuse, behavioral factors (physical inactivity, smoking status, number of alcohol drinks per week) during follow-up, anxiety disorders and illicit drug dependence during follow-up, possibly weight gain inducing medication (other than antidepressants) during follow-up, and length of follow-up, major depressive disorder (MDD) subtypes at baseline and during follow-up and current vs. remitted MDD status at follow-up, severity during follow-up (number of symptoms of most severe major depressive episode (MDE), time spent in MDE, global assessment functioning (GAF) score, suicidality, hospitalization, psychotic features), and relatives with MDD.*

**Table S7: Participants with 5% adiposity markers increase during follow-up by any antidepressant use prior to baseline and during follow-up**

|  | **Change (5% increase) in adiposity markers during follow-up** | | | | | | | | |
| --- | --- | --- | --- | --- | --- | --- | --- | --- | --- |
|  | **Crude change** | | | **Model 1** | | **Model 2** | | **Model 3** | |
|  | **%** | **OR^1^** | **(95%CI)** | **OR** | **(95%CI)** | **OR** | **(95%CI)** | **OR** | **(95%CI)** |
| **Body Mass Index [kg/m^2^] (n=2462)** |  |  |  |  |  |  |  |  |  |
| Antidepressants prior to baseline |  |  |  |  |  |  |  |  |  |
| Number of compounds | - | 0.99 | (0.91,1.08) | 0.91 | (0.83,1.01) | **0.88*** | **(0.79,0.97)** | **0.88*** | **(0.79,0.97)** |
| Antidepressants during follow-up |  |  |  |  |  |  |  |  |  |
| Any antidepressants | 41.7 | **1.64***** | **(1.31,2.05)** | **1.75***** | **(1.36,2.25)** | **1.72***** | **(1,31,2.25)** | **1.67***** | **(1.27,2.19)** |
| No antidepressants (ref.) | 29.3 | 1 (ref.) | - | 1 (ref.) | - | 1 (ref.) | - | 1 (ref.) | - |
| **Waist circumference [cm] (n=2475)** |  |  |  |  |  |  |  |  |  |
| Antidepressants prior to baseline |  |  |  |  |  |  |  |  |  |
| Number of compounds | - | 1.03 | (0.95,1.12) | **0.90*** | **(0.82,0.98)** | **0.88**** | **(0.80,0.97)** | **0.88**** | **(0.80,0.97)** |
| Antidepressants during follow-up |  |  |  |  |  |  |  |  |  |
| Any antidepressants | 53.2 | **1.43**** | **(1.15,1.78)** | **1.58***** | **(1.23,2.03)** | **1.51**** | **(1.16,1.96)** | **1.51**** | **(1.16,1.98)** |
| No antidepressants (ref.) | 40.9 | 1 (ref.) | - | 1 (ref.) | - | 1 (ref.) | - | 1 (ref.) | - |
| **Fat mass [%] (n=2079)** |  |  |  |  |  |  |  |  |  |
| Antidepressants prior to baseline |  |  |  |  |  |  |  |  |  |
| Number of compounds | - | 1.02 | (0.93,1.11) | 0.97 | (0.88,1.07) | 0.95 | (0.86,1.05) | 0.95 | (0.86,1.06) |
| Antidepressants during follow-up |  |  |  |  |  |  |  |  |  |
| Any antidepressants | 62.6 | 1.07 | (0.84,1.37) | 1.08 | (0.82,1.42) | 1.03 | (0.77,1.38) | 1.03 | (0.77,1.39) |
| No antidepressants (ref.) | 61.4 | 1 (ref.) | - | 1 (ref.) | - | 1 (ref.) | - | 1 (ref.) | - |

OR: odd ratio; 95%CI: 95% confidence interval. ref.: reference group. * p<0.05, ** p<0.01, *** p<0.001.

Model 1 adjusted for socio-demographic characteristics (sex, age, socio-economic status, living alone during follow-up), early physical and sexual abuse, behavioral factors (physical inactivity, smoking status, number of alcohol drinks per week) during follow-up, anxiety disorders and illicit drug dependence during follow-up, possibly weight gain inducing medication (other than antidepressants) during follow-up, and length of follow-up.

Model 2 = model 1 additionally adjusted for major depressive disorder (MDD) subtypes at baseline and during follow-up and current vs. remitted MDD status at follow-up.

Model 3 = model 2 additionally adjusted for severity during follow-up (number of symptoms of most severe major depressive episode (MDE), time spent in MDE, global assessment functioning (GAF) score, suicidality, hospitalization, psychotic features), and relatives with MDD.

^1^ Each model adjusted for age, sex.

**Table S8: Participants with 5% adiposity markers increase during follow-up by use of antidepressant class prior to baseline and during follow-up**

|  | **Change (5% increase) in adiposity markers during follow-up** | | | | | | | | |
| --- | --- | --- | --- | --- | --- | --- | --- | --- | --- |
|  | **Crude change** | | | **Model 1** | | **Model 2** | | **Model 3** | |
|  | **%** | **OR^1^** | **(95%CI)** | **OR** | **(95%CI)** | **OR** | **(95%CI)** | **OR** | **(95%CI)** |
| **Body Mass Index [kg/m^2^] (n=2462)** |  |  |  |  |  |  |  |  |  |
| Antidepressants prior to baseline |  |  |  |  |  |  |  |  |  |
| TCA | 25.7 | 0.77 | (0.46,1.32) | **0.54*** | **(0.30,1.00)** | **0.51*** | **(0.28,0.94)** | **0.51*** | **(0.27,0.95)** |
| SSRI | 35.7 | 1.16 | (0.89,1.52) | 0.89 | (0.65,1.21) | 0.77 | (0.56,1.07) | 0.79 | (0.57,1.09) |
| Mirtazapine/Trazodone | 29.2 | 0.89 | (0.37,2.17) | 0.84 | (0.32,2.15) | 0.78 | (0.30,2.01) | 0.72 | (0.27,1.89) |
| Other^2^ | 34.2 | 1.04 | (0.54,2.01) | 0.71 | (0.34,1.47) | 0.71 | (0.34,1.49) | 0.74 | (0.35,1.56) |
| No antidepressants (ref.) | 30.9 | 1 (ref.) | - | 1 (ref.) | - | 1 (ref.) | - | 1 (ref.) | - |
| Antidepressants during follow-up |  |  |  |  |  |  |  |  |  |
| TCA | 44.2 | **1.81*** | **(1.03,3.16)** | **1.94*** | **(1.03,3.65)** | 1.84 | (0.97,3.49) | 1.80 | (0.94,3.45) |
| SSRI | 43.3 | **1.68***** | **(1.30,2.18)** | **1.65**** | **(1.22,2.23)** | **1.68**** | **(1.23,2.31)** | **1.63**** | **(1.18,2.25)** |
| Mirtazapine/ Trazodone | 31.9 | 0.99 | (0.53,1.85) | 0.85 | (0.44,1.65) | 0.90 | (0.46,1.77) | 0.89 | (0.45,1.75) |
| Other^2^ | 43.9 | **1.66*** | **(1.06,2.60)** | 1.67 | (1.00,2.79) | 1.62 | (0.95,2.75) | 1.51 | (0.88,2.60) |
| No antidepressants (ref.) | 29.3 | 1 (ref.) | - | 1 (ref.) | - | 1 (ref.) | - | 1 (ref.) | - |
| **Waist circumference [cm] (n=2475)** |  |  |  |  |  |  |  |  |  |
| Antidepressants prior to baseline |  |  |  |  |  |  |  |  |  |
| TCA | 48.7 | 1.21 | (0.76,1.94) | 0.84 | (0.50,1.43) | 0.82 | (0.48,1.40) | 0.82 | (0.48,1.40) |
| SSRI | 45.7 | 0.92 | (0.71,1.19) | **0.73*** | **(0.54,0.99)** | **0.70*** | **(0.51,0.95)** | **0.70*** | **(0.52,0.96)** |
| Mirtazapine/Trazodone | 29.2 | 0.53 | (0.22,1.32) | 0.46 | (0.18,1.19) | 0.45 | (0.17,1.16) | 0.42 | (0.16,1.11) |
| Other^2^ | 48.8 | 1.06 | (0.56,2.00) | 0.85 | (0.42,1.73) | 0.85 | (0.42,1.73) | 0.87 | (0.42,1.78) |
| No antidepressants (ref.) | 42.3 | 1 (ref.) | - | 1 (ref.) | - | 1 (ref.) | - | 1 (ref.) | - |
| Antidepressants during follow-up |  |  |  |  |  |  |  |  |  |
| TCA | 69.8 | **3.14***** | **(1.71,5.76)** | **3.36***** | **(1.73,6.50)** | **3.24***** | **(1.67,6.30)** | **3.22***** | **(1.66,6.27)** |
| SSRI | 53.4 | **1.36*** | **(1.05,1.77)** | **1.48*** | **(1.09,2.00)** | **1.42*** | **(1.04,1.94)** | **1.42*** | **(1.03,1.96)** |
| Mirtazapine/ Trazodone | 42.6 | 0.91 | (0.50,1.67) | 0.85 | (0.45,1.62) | 0.83 | (0.44,1.59) | 0.83 | (0.43,1.60) |
| Other^2^ | 53.7 | 1.42 | (0.90,2.23) | 1.37 | (0.81,2.30) | 1.29 | (0.76,2.19) | 1.27 | (0.74,2.18) |
| No antidepressants (ref.) | 40.9 | 1 (ref.) | - | 1 (ref.) | - | 1 (ref.) | - | 1 (ref.) | - |
| **Fat mass [%] (n=2079)** |  |  |  |  |  |  |  |  |  |
| Antidepressants prior to baseline |  |  |  |  |  |  |  |  |  |
| TCA | 57.8 | 0.90 | (0.54,1.50) | 0.89 | (0.51,1.55) | 0.86 | (0.49,1.51) | 0.89 | (0.51,1.57) |
| SSRI | 62.3 | 1.06 | (0.80,1.41) | 1.01 | (0.73,1.40) | 0.97 | (0.69,1.35) | 0.98 | (0.70,1.37) |
| Mirtazapine/Trazodone | 55.6 | 0.79 | (0.31,2.00) | 0.81 | (0.30,2.14) | 0.81 | (0.31,2.16) | 0.77 | (0.29,2.06) |
| Other^2^ | 59.5 | 0.93 | (0.48,1.80) | 0.88 | (0.42,1.84) | 0.86 | (0.41,1.80) | 0.84 | (0.40,1.76) |
| No antidepressants (ref.) | 62.1 | 1 (ref.) | - | 1 (ref.) | - | 1 (ref.) | - | 1 (ref.) | - |
| Antidepressants during follow-up |  |  |  |  |  |  |  |  |  |
| TCA | 61.7 | 1.05 | (0.58,1.91) | 1.09 | (0.57,2.11) | 1.05 | (0.54,2.03) | 1.02 | (0.53,1.99) |
| SSRI | 64.3 | 1.15 | (0.86,1.54) | 1.12 | (0.80,1.57) | 1.11 | (0.78,1.56) | 1.13 | (0.79,1.61) |
| Mirtazapine/Trazodone | 63.9 | 1.10 | (0.55,2.18) | 1.07 | (0.52,2.17) | 1.05 | (0.51,2.15) | 1.07 | (0.52,2.21) |
| Other^2^ | 62.3 | 1.03 | (0.65,1.65) | 1.00 | (0.58,1.71) | 0.94 | (0.55,1.63) | 0.95 | (0.54,1.66) |
| No antidepressants (ref.) | 61.4 | 1 (ref.) | - | 1 (ref.) | - | 1 (ref.) | - | 1 (ref.) | - |

TCA: Tricyclic antidepressants; SSRI: Selective Serotonin Reuptake Inhibitors; OR: odd ratio; 95%CI: 95% confidence interval. ref.: reference group. * p<0.05, ** p<0.01, *** p<0.001.

Model 1 adjusted for socio-demographic characteristics (sex, age, socio-economic status, living alone during follow-up), early physical and sexual abuse, behavioral factors (physical inactivity, smoking status, number of drinks per week) during follow-up, anxiety disorders and illicit drug dependence during follow-up, possibly weight gain inducing medication (other than antidepressants) during follow-up, and length of follow-up.

Model 2 = model 1 additionally adjusted for major depressive disorder (MDD) subtypes at baseline and during follow-up and current vs. remitted MDD status at follow-up.

Model 3 = model 2 additionally adjusted for severity during follow-up (number of symptoms of most severe major depressive episode (MDE), time spent in MDE, global assessment functioning (GAF) score, suicidality, hospitalization, psychotic features), and relatives with MDD.

^1^ Each model adjusted for age, sex.

^2^ SNRI (Serotonin-Noradrenaline Reuptake Inhibitors)/NDRI (Noradrenaline Reuptake Inhibitors)/MAOI(Monoamine Oxidase Inhibitors).

**Table S9:** **Participants with 5% adiposity markers increase during follow-up by use of specific antidepressants during follow-up**

|  | **Change (5% increase) in adiposity markers during follow-up** | | | | | | | | |
| --- | --- | --- | --- | --- | --- | --- | --- | --- | --- |
|  | **Body Mass Index [kg/m^2^]**  **(n=2462)** | | | **Waist circumference [cm]**  **(n=2475)** | | | **Fat mass [%]**  **(n=2079)** | | |
|  | **%** | **OR^1^** | **(95%CI)** | **%** | **OR^1^** | **(95%CI)** | **%** | **OR^1^** | **(95%CI)** |
| TCA |  |  |  |  |  |  |  |  |  |
| Clomipramine | 20.0 | 0.29 | (0.05,1.65) | 45.4 | 0.67 | (0.17,2.57) | 50.0 | 0.51 | (0.13,2.07) |
| Amitriptyline | 40.0 | 1.05 | (0.40,2.74) | 70.0 | **3.08*** | **(1.12,8.49)** | 57.9 | 0.78 | (0.30,2.04) |
| Melitracen and psycholeptics | 50.0 | 1.73 | (0.59,5.08) | 75.0 | 3.11 | (0.93,10.37) | 84.6 | 3.22 | (0.68,15.34) |
| SSRI |  |  |  |  |  |  |  |  |  |
| Fluoxetine | 41.3 | 1.20 | (0.71,2.00) | 63.2 | 1.59 | (0.95,2.67) | 65.6 | 1.26 | (0.72,2.21) |
| Citalopram | 35.1 | 0.81 | (0.48,1.37) | 51.9 | 1.07 | (0.65,1.76) | 66.1 | 1.14 | (0.64,2.04) |
| Paroxetine | 35.0 | 1.04 | (0.52,2.10) | 40.0 | 0.65 | (0.33,1.30) | 68.8 | 1.46 | (0.66,3.24) |
| Sertraline | 51.6 | 2.08 | (0.98,4.41) | 50.0 | 1.19 | (0.57,2.48) | 74.1 | 1.62 | (0.67,3.94) |
| Escitalopram | 51.7 | **2.31***** | **(1.43,3.72)** | 59.8 | **1.79*** | **(1.10,2.91)** | 62.7 | 0.96 | (0.56,1.66) |
| Mirtazapine/Trazodone |  |  |  |  |  |  |  |  |  |
| Mirtazapine | 25.8 | 0.59 | (0.24,1.42) | 35.5 | 0.56 | (0.24,1.30) | 72.7 | 1.48 | (0.55,4.04) |
| Trazodone | 42.8 | 1.43 | (0.50,4.09) | 56.3 | 1.23 | (0.42,3.62) | 50.0 | 0.62 | (0.20,1.85) |
| SNRI |  |  |  |  |  |  |  |  |  |
| Venlafaxine | 42.0 | 1.22 | (0.64,2.31) | 52.0 | 1.03 | (0.55,1.95) | 58.7 | 0.77 | (0.40,1.47) |
| Duloxetine | 48.5 | 1.73 | (0.81,3.70) | 63.9 | 1.93 | (0.87,4.27) | 67.4 | 1.31 | (0.58,2.99) |
| No antidepressants (ref.) | 29.3 | 1 (ref.) | - | 40.9 | 1 (ref.) | - | 61.4 | 1 (ref.) | - |

TCA: tricyclic antidepressants; SSRI: selective serotonin reuptake inhibitors; SNRI: serotonin-noradrenaline reuptake inhibitors; OR: odd ratio; 95%CI: 95% confidence interval. ref.: reference group. * p<0.05, ** p<0.01, *** p<0.001.

^1^ Adjusted for socio-demographic characteristics, early physical and sexual abuse, behavioral factors during follow-up, anxiety disorders and illicit drug dependence during follow-up, possibly weight gain inducing medication (other than antidepressants) during follow-up, length of follow-up, major depressive disorder (MDD) subtypes at baseline and during follow-up and current vs. remitted MDD status at follow-up, severity during follow-up (number of symptoms of most severe major depressive episode (MDE), time spent in MDE, global assessment functioning (GAF) score, suicidality, hospitalization, psychotic features), relatives with MDD, number of different antidepressant compounds prior to baseline, and other antidepressants (Fluvoxamine, Bupropion, Reboxetine, Moclobémide, Trimipramine, Mianserin) during follow-up.

**Figure S1: Flow chart of the study sample selection**

CoLaus (physical exam) baseline

Age 35-66 years

n = 5535

PsyCoLaus (psychiatric exam) baseline

n = 3719

Missing MDD diagnosis at baseline n = 6

Bipolar or schizoaffective disorder, schizophrenia or eating disorder at baseline n = 153

Non-Caucasians

n = 290

Baseline sample

n = 3270

Deaths

n = 43

No participation at CoLaus follow-up n = 414

CoLaus follow-up sample

n = 2813

No participation at PsyCoLaus follow-up n = 324

Missing information on change in all adiposity markers n = 10

Final follow-up sample

n = 2479

Missing information on BMI n = 17

Missing information on waist n = 4

Missing information on fat mass n = 400

Waist circumference n = 2475

Fat

mass

n = 2079

Body mass index

n = 2462

Key: MDD: Major depressive disorder, BMI: Body mass index

= Baseline assessment

= Follow-up assessment

**Figure S2: Data assessments for the analyses of associations between antidepressant use and change in adiposity markers**


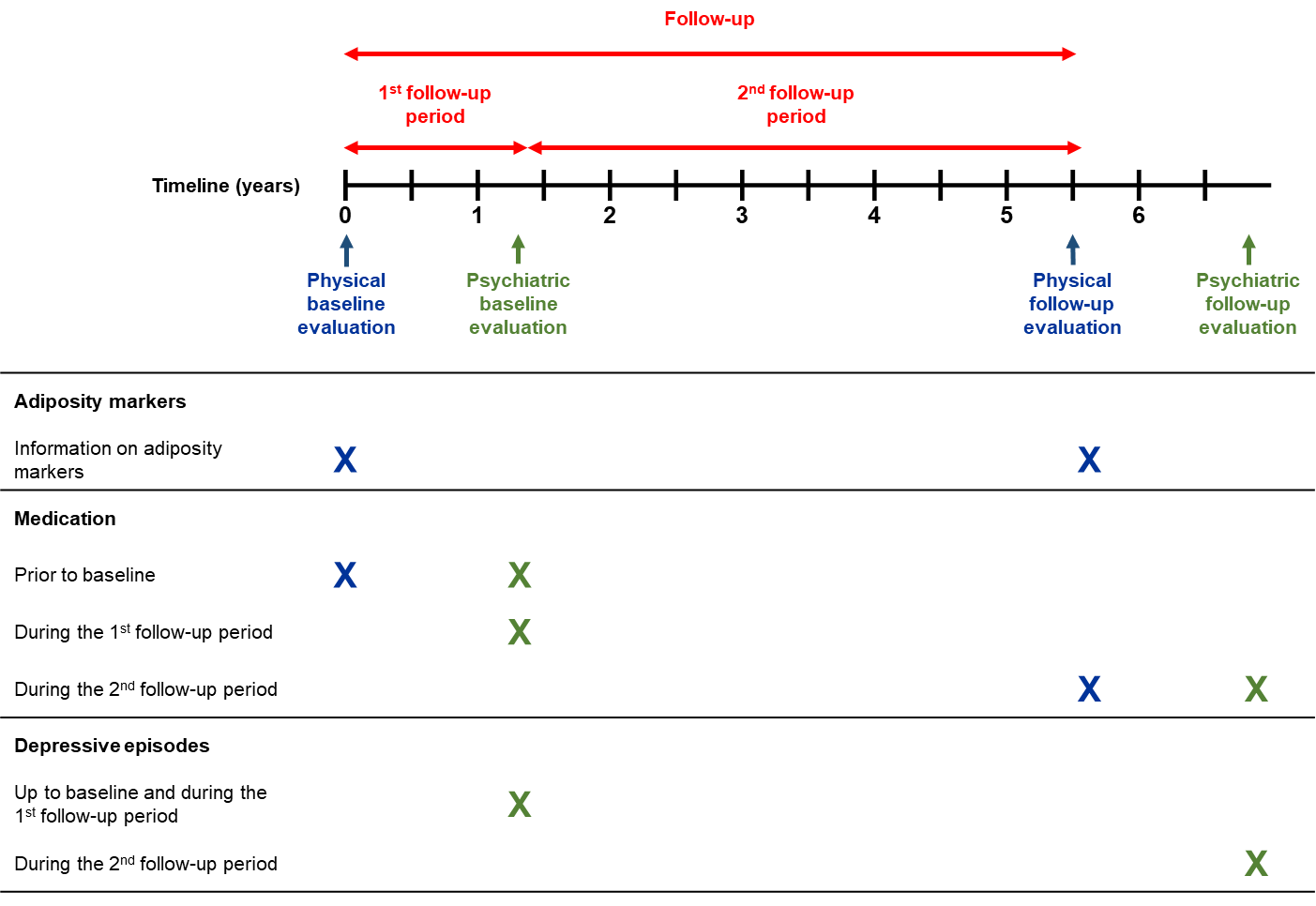

Supplement: Supplementary file 1 — Supplementary information [file 41398_2024_3032_MOESM1_ESM.docx]
